# Adipocyte‐Derived Exosomal MTTP Suppresses Ferroptosis and Promotes Chemoresistance in Colorectal Cancer

**DOI:** 10.1002/advs.202203357

**Published:** 2022-08-17

**Authors:** Qiumo Zhang, Ting Deng, Hongdian Zhang, Duo Zuo, Qihang Zhu, Ming Bai, Rui Liu, Tao Ning, Le Zhang, Zhentao Yu, Haiyang Zhang, Yi Ba

**Affiliations:** ^1^ Tianjin Medical University Cancer Institute and Hospital National Clinical Research Center for Cancer Key Laboratory of Cancer Prevention and Therapy Tianjin's Clinical Research Center for Cancer Tianjin 300060 China; ^2^ Department of Thoracic Surgery National Cancer Center National Clinical Research Center for Cancer Cancer Hospital & Shenzhen Hospital Chinese Academy of Medical Sciences and Peking Union Medical College Shenzhen 518172 China

**Keywords:** adipose tissue, chemoresistance, colorectal cancer, exosomes, ferroptosis, MTTP, organoids

## Abstract

Obesity is closely related to a poor prognosis in patients with advanced colorectal cancer (CRC), but the mechanisms remain unclear. Ferroptosis is a form of nonapoptotic cell death characterized by lipid reactive oxygen species (ROS) accumulation and iron dependency and is associated with the chemoresistance of tumors. Here, it is shown that adipose‐derived exosomes reduce ferroptosis susceptibility in CRC, thus promoting chemoresistance to oxaliplatin. It is found that microsomal triglyceride transfer protein (MTTP) expression is increased in the plasma exosomes of CRC patients with a high body fat ratio, serving as an inhibitor of ferroptosis and reducing sensitivity to chemotherapy. Mechanistically, the MTTP/proline‐rich acidic protein 1 (PRAP1) complex inhibited zinc finger E‐box binding homeobox 1 expression and upregulated glutathione peroxidase 4 and xCT, leading to a decreased polyunsaturated fatty acids ratio and lipid ROS levels. Moreover, experiments are carried out in organoids, and a tumor implantation model is established in obese mice, demonstrating that the inhibition of MTTP increases the sensitivity to chemotherapy. The results reveal a novel intracellular signaling pathway mediated by adipose‐derived exosomes and suggest that treatments targeting secreted MTTP might reverse oxaliplatin resistance in CRC.

## Graphical abstract

The authors show that adipose‐derived exosomes reduce ferroptosis susceptibility in CRC, thus promoting chemoresistance to oxaliplatin. MTTP expression is increased in the plasma exosomes of CRC patients with a high body fat ratio, leading to a decreased polyunsaturated fatty acid (PUFA) ratio and lipid ROS levels, which serving as an inhibitor of ferroptosis and reducing sensitivity to chemotherapy.

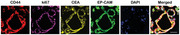

## 1 Introduction

Colorectal cancer (CRC) is the second most common cancer worldwide and has increasing morbidity and mortality rates.^[^ ^1^ ^]^ Annually, CRC accounts for more than 1 350 000 new cancer cases and ≈700 000 deaths.^[^ ^2^ ^]^ As recommended by several treatment guidelines, adjuvant oxaliplatin‐based chemotherapy is the standard of care for patients with advanced CRC.^[^ ^3^, ^4^ ^]^ Conventional chemotherapy exerts limited effects on late‐stage CRC, and chemoresistance is one of the main causes of a poor prognosis.^[^ ^5^ ^]^ Chemotherapy resistance is generally associated with complex causes, including reduced cellular drug accumulation, increased activity of the detoxification system, an increased damaged DNA repair process and inhibition of pathways that promote cell death.^[^ ^6^ ^]^ Hence, an understanding of the molecular mechanisms is urgently needed to develop new strategies for the treatment of CRC.

Substantial evidence shows that obesity contributes to 20% of cancer‐related deaths and is associated with an increased risk of CRC.^[^ ^7^, ^8^ ^]^ Compared to normal weight status, stage II and III obesity are significantly associated with shorter overall survival in patients with CRC.^[^ ^9^ ^]^ Obese individuals are estimated to have a 33% higher CRC risk and worse CRC prognosis than those with a normal body mass index (BMI).^[^ ^9^ ^]^ Recent studies have indicated that obesity promotes chemoresistance by inducing chronic inflammation, altering pharmacokinetics, and altering tumor‐associated adipokine secretion.^[^ ^10^ ^]^

Ferroptosis is an iron‐dependent form of nonapoptotic cell death that is characterized by the accumulation of cytotoxic lipid reactive oxygen species (ROS) and results in lipid membrane damage and perforation.^[^ ^11^, ^12^ ^]^ Activation of ferroptosis has been reported to contribute to the efficacy of cancer treatments, such as immune checkpoint blockade,^[^ ^14^ ^]^ radiotherapy^[^ ^15^ ^]^ and chemotherapy.^[^ ^13^ ^]^ The classic cellular defense mechanism against ferroptosis is achieved by the glutathione peroxidase 4 (GPX4)‐xCT signaling axis. GPX4, a glutathione peroxidase, converts lipid hydroperoxides into lipid alcohols and thereby suppresses lipid peroxidation and inhibits ferroptosis.^[^ ^14^, ^15^, ^16^ ^]^ RSL3, a specific GPX4 inhibitor, induces ROS accumulation in CRC cells and ultimately leads to ferroptosis.^[^ ^17^ ^]^ xCT is responsible for importing cystine, and cystine is then reduced to cysteine, which is the raw material for glutathione synthesis.^[^ ^18^, ^19^, ^20^ ^]^ Correspondingly, inactivation of GPX4 or xCT by genetic or pharmacological approaches typically induces ferroptosis.^[^ ^11^ ^]^ The deubiquitylase OTUB1 plays an essential role in regulating the stability of SLC7A11 and CD44‐mediated ferroptosis in human cancers.^[^ ^21^ ^]^ Furthermore, targeting SLC7A11 could specifically induce ferroptosis and suppress the progression of CRC stem cells.^[^ ^22^ ^]^ BAP1 induces ferroptosis and suppresses tumor development by repressing SLC7A11 expression.^[^ ^23^ ^]^ Lipid peroxidation plays a vital role in ferroptosis and is dependent on the activity of acyl‐CoA synthetase long‐chain family member 4 (ACSL4),^[^ ^24^, ^25^ ^]^ which is required for polyunsaturated fatty acid (PUFA)‐phospholipid (PL) biosynthesis and is characterized by the preferential utilization of arachidonic acid (AA)^[^ ^23^ ^]^ and phospholipid ethanolamine (PE).^[^ ^25^, ^26^, ^27^, ^28^ ^]^ Studies have shown that both types of PUFAs are abundant in intestinal epithelial cells.^[^ ^29^ ^]^

Since ferroptosis is caused by the peroxidation of PUFAs, the regulation of lipid metabolism is a crucial determinant in the susceptibility to ferroptosis. However, the potential roles of adipocyte exosomes in regulating lipid metabolism and ferroptosis in CRC cells remain unknown. Microsomal triglyceride transfer protein (MTTP) is highly expressed in adipose tissue and is involved in regulating lipid metabolism by facilitating triglyceride transport between membrane vesicles,^[^ ^30^ ^]^ which is closely linked to the mitigation of ferroptosis. Proline‐rich acidic protein 1 (PRAP1) colocalizes with MTTP in the ER and facilitates MTTP‐mediated lipid transport.^[^ ^31^ ^]^ PRAP1 protects gastrointestinal epithelial cells from radiation‐induced apoptosis.^[^ ^32^ ^]^ Therapy‐resistant cancer cells have recently been reported to exhibit increased susceptibility to ferroptosis,^[^ ^33^, ^34^ ^]^ possibly due to their increased lipid metabolism, which is mediated by zinc finger E‐box binding homeobox 1 (ZEB1).^[^ ^35^ ^]^

In the present study, we assessed whether MTTP was overexpressed in the plasma exosomes of patients with CRC presenting a high body fat ratio. We found that the exosomal MTTP/PRAP1/ZEB1 axis inhibited lipid ROS production and decreased PUFA levels by upregulating GPX4 and xCT. Moreover, chemotoxicity promoted MTTP expression and secretion from adipocytes, which suppressed ferroptosis in CRC cells and resulted in acquired chemoresistance. Our results provide evidence that a high body fat ratio is associated with a high risk of CRC and suggest that approaches targeting adipose‐derived MTTP represent a new strategy for the clinical treatment of CRC patients.

## 2 Results

### 2.1 Characteristics of Serum Exosome Protein Expression in High Body Fat Patients with CRC

**Figure 1:**
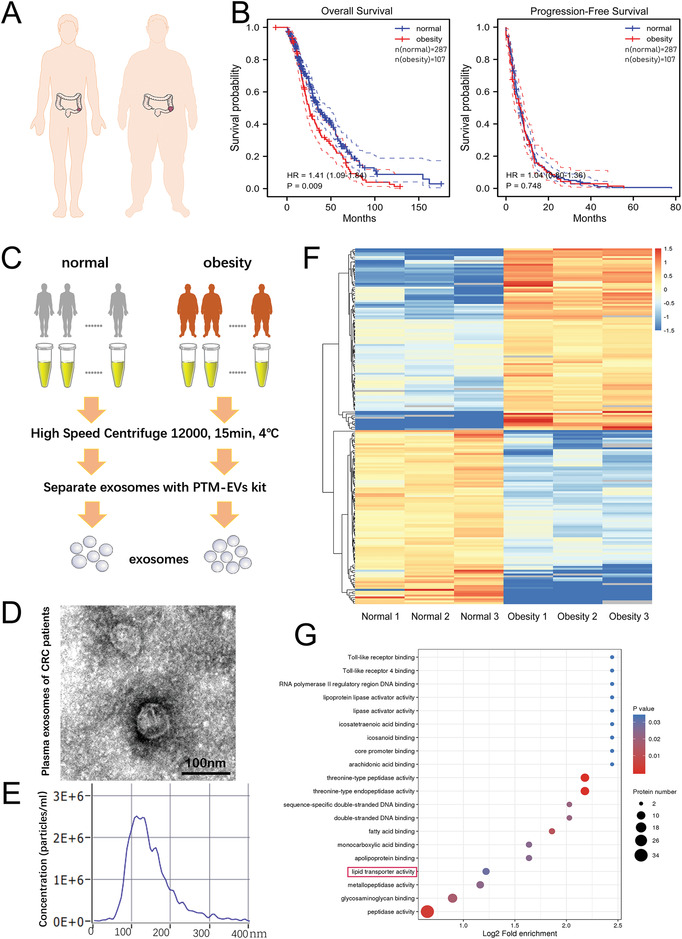
Characteristics of serum exosome protein expression in high body fat patients with CRC: A) Schematic diagram of patients with CRC presenting normal body fat and high body fat. B) Overall survival and progression‐free survival of patients with normal body fat and high body fat who were treated at Tianjin Medical University Cancer Institute and Hospital. C) Flow chart of methods used to collect plasma and separate plasma exosomes from patients with CRC. D) TEM image of exosomes isolated from human plasma (scale bar, 100 nm). E) Size range of the plasma exosomes detected using NTA. (F) Heatmap of plasma exosomal proteins that were differentially expressed in normal and obese patients. G) KEGG pathway enrichment analysis of plasma from obese patients compared with control normal weight patients.

Obesity is recognized as a negative prognostic factor for colorectal cancer, with patients having a 50% increased risk of developing CRC and a 30% higher risk of dying from CRC.^[^ ^37^ ^]^ Overweight and obesity are associated with an increased risk of T3 or T4 tumors and N1 or N2 lymph node staging in colon cancer.^[^ ^38^ ^]^ Moreover, among patients who receive treatment for CRC, obese patients experience shorter overall survival than normal weight patients.^[^ ^39^ ^]^ We retrospectively analyzed the survival and prognosis of all patients (n = 394) with advanced colorectal cancer over 5 years (2014.6‐2019.6) at Tianjin Medical University Cancer Institute and Hospital. All patients information were listed in **Table** **1**. The body fat ratio of female was calculated as: 64.8 − 752 × (1/BMI) + 0.016 × age, of male was calculated as: 51.9 − 740 × (1/BMI) + 0.029 × age.^[^ ^40^ ^]^ Then, the body fat ratio of patients were compared with table in Figure S1C (Supporting Information) to define whether the patient was obese. The effect of body fat percentage on survival was analyzed, and patients with normal body fat had a better clinical outcome than those obesity, with an improvement in overall survival (OS) (34.9 m vs 25.3 m) but not in progression‐free survival (PFS) (6.5 m vs 7 m) (**Figure** **1**B). The cellular mechanisms by which obesity negatively affects chemotherapy outcomes must be explored. Thus, we performed an LC–MS/MS analysis of plasma exosomes from obese (*n* = 45) and normal‐weight (*n* = 30) patients with CRC (**Table** **2**, Figure S1B, Supporting Information). The workflow for the isolation of exosomes is illustrated in Figure 1C. Exosomes were isolated from human plasma samples through ultrahigh‐speed centrifugation and observed to have a typical round morphology (Figure 1D). The diameter of exosomes from CRC patients were shown in Figure 1E. It has been reported that the concentration of circulating exosome was significantly higher in obese individuals than in control ones,^[^ ^41^ ^]^ which is consistent with our findings (Figure S1C, Supporting Information). We performed a comparative proteomics analysis of exosomes derived from the plasma of patients with advanced CRC presenting a high body fat and normal weight using mass spectrometry to explore the underlying mechanisms. Overall, 1011 unique proteins were identified and quantified in both groups. Most of the significantly altered proteins, including 95 upregulated and 91 downregulated proteins in the obesity group compared with the normal weight group (fold change > 1.5), showed good consistency in each sample (Figure 1F). In GO classification analysis, metabolic process was one of the most significantly upregulated biological process terms (Figure S1D, Supporting Information). In KEGG pathway enrichment analysis, lipoprotein and lipid transport‐related pathways were the most significantly upregulated metabolic pathways (Figure 1G, Figure S1E, Supporting Information). The above evidence suggests that high body fat is associated with a poor prognosis for patients with CRC cancer.

**Table 1:** Body fat ratio and clinicopathological features of advanced CRC patients (*n* = 394)

Clinical characteristic	Body fat			
	Normal	Obesity	Total	*P* value
Overall	287	107	394	
Gender				
Female	83 (21.1%)	70 (17.8%)	153	<0.001*
Male	204 (51.8%)	37 (9.4%)	241	
T stage				
T1–T2	43 (11.7%)	3 (0.8%)	46	0.008*
T3–T4	226 (61.5%)	95 (25.9%)	321	
N stage				
N0	88 (24%)	19 (5.2%)	107	<0.001*
N1 + N2 + N3	181 (49.3%)	79 (21.4%)	260	
M stage				
M0	32 (8.7%)	133 (35.8%)	165	0.008*
M1	69 (18.6%)	137 (36.9%)	206	
Clinical stage				
I + II	27 (7%)	3 (0.8%)	30	0.001*
III + IV	254 (65.6%)	104 (26.6%)	358	
Age, median (IQR)	60 (54, 66)	61 (56, 65.5)		0.545

Note: The result was analyzed by the Pearson chi‐square test. *P* values with significance were shown as asterisk.

* *P* < 0.05.

**Table 2:** Body fat ratio and clinicopathological features of advanced CRC patients whose plasma was collected for LC‐MS/MS analysis (*n* = 75)

Clinical characteristic	Body fat			
	Normal	Obesity	Total	*P* value
Overall	30	45	75	
Gender				
Female	1 (1.3%)	43 (57.3%)	44	<0.001*
Male	29 (38.7%)	2 (2.7%)	31	
T stage				
T2	7 (10.6%)	6 (9.1%)	13	
T3	9 (13.6%)	10 (15.2%)	19	0.111
T4	13 (19.6%)	21 (31.8%)	34	
N stage				
N0	7 (13.2%)	2 (3.8%)	9	
N1	6 (11.3%)	12 (22.6%)	18	0.038*
N2	12 (22.7%)	14 (24.5%)	26	
M stage				
M0	4 (5.3%)	5 (6.7%)	9	1
M1	26 (34.7%)	40 (53.3%)	66	
Clinical stage				
II	1 (1.3%)	0 (0%)	1	
III	2 (2.7%)	3 (4%)	5	0.634
IV	27 (36%)	42 (56%)	69	
Age, median (IQR)	55 (35.25, 58)	62 (56, 67)		< 0.001*

Note: The result was analyzed by the Pearson chi‐square test. *P* values with significance were shown as asterisk.

* *P* < 0.05.

### 2.2 GPX4 and xCT are Expressed at High Levels in CRC and Associated with a Poor Prognosis

First, we performed cell viability assays using HCT116 and SW480 cell lines. We treated these cell lines individually with erastin (6.25–100 × 10^−6^ m) for 48 h and then observed reduced viability of the two cell lines in a dose‐dependent manner, which was partially restored by the ferroptosis inhibitor liproxstatin‐1 (Lip‐1) rather than by the apoptosis inhibitor Z‐VAD‐FMK (ZVAD), the necroptosis inhibitor necrostatin‐1s (Nec‐1) or the autophagy blocker chloroquine (CQ) (Figure S2A, Supporting Information). Since drug resistance is associated with reactive oxygen species (ROS) production^[^ ^43^, ^44^ ^]^ and ferroptosis is driven by lipid peroxidation,^[^ ^45^ ^]^ which is a type of ROS, we observed (using C11‐BODIPY staining, details are provided in the Materials and methods) that erastin and oxaliplatin (L‐OHP) induced lipid peroxidation in CRC cells (Figure S2B, Supporting Information). Both erastin and L‐OHP treatment caused the generation of a significant amount of lipid ROS. GPX4 and xCT are two key components involved in ferroptosis pathways that function to inhibit ferroptosis.^[^ ^16^, ^17^ ^]^ Thus, the expression levels of GPX4 and SLC7A11 in patients with CRC were analyzed using GEPIA (http://gepia.cancer‐pku.cn), an open access database. TCGA and GTEx data revealed high GPX4 and SLC7A11 expression in colorectal cancer (Figure S2C, Supporting Information). The Kaplan–Meier survival curves predicted the prognosis of patients with CRC. Briefly, during follow‐up, the survival rate of the high GPX4 expression group was consistently lower than that of the low GPX4 expression group, suggesting that high GPX4 expression was significantly correlated with shorter overall survival (OS) of patients with CRC (Figure S2D, Supporting Information). We then examined the expression levels of GPX4 and xCT in response to treatment with erastin. As shown in Figure S2E (Supporting Information), the expression of both xCT and GPX4 in CRC cells was decreased after the addition of erastin in a dose‐dependent manner. Then, we chose 8 paired serial sections of cancerous and adjacent noncancerous tissues from patients with CRC to assess the expression levels of GPX4 and xCT. As expected, high levels of GPX4 and xCT expression were clearly observed in tumor tissues compared with noncancerous tissues (Figure S2F, Supporting Information).

### 2.3 In Vitro Coculture Model of Adipose Exosomes and CRC Cells

**Figure 2:**
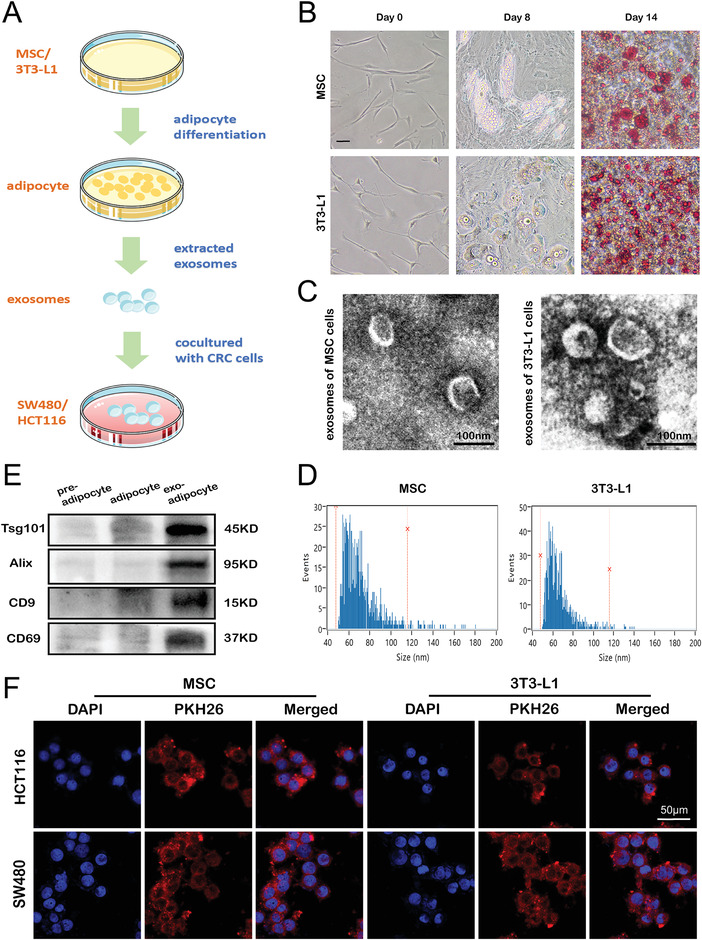
Coculture model of adipose exosomes and CRC cells: A) Schematic diagram of adipocyte differentiation, exosome extraction and coculture of adipocyte exosomes with CRC cells. B) Photos of MSC/3T3‐L1 and MSC/3T3‐L1 cells induced to differentiate for 8 days, as well as Oil red O staining of mature adipocytes. C) Transmission electron microscopy image of MSC/3T3‐L1 exosomes (scale bar, 100 nm). D) Particle size distribution of MSC/3T3‐L1exosomes measured by nanoflow cytometer. E) Tsg101, Alix, CD9 and CD69 expression, as detected using WB. F) PKH26‐labeled MSC/3T3‐L1 exosomes were taken up by HCT116/SW480 cells.

As described in **Figure** **2**A, preadipocytes were induced to differentiate into mature adipocytes for 14 days. Undifferentiated MSC and 3T3‐L1 cells were spindle‐shaped. After differentiation induction for 8 days, the cells became larger and rounder, and transparent lipid droplets with ring shapes were observed around the nucleus. Mature adipocytes formed at day 14 and were stained red with Oil Red O (Figure 2B). Then, exosomes were isolated from mature adipocyte culture medium by gradient differential centrifugation. The morphology of exosomes was photographed via a transmission electron microscope (TEM) (Figure 2C). The diameter of exosomes was analyzed via nanoflow cytometer, the mean size of adipocyte exosomes was 69.6 nm (from MSC) and 65.6 nm (from 3T3‐L1) (Figure 2D). Western blots were performed to characterize the marker proteins of exosomes and revealed the highest levels in exosomes derived from mature adipocyte, followed by mature adipocytes, and the lowest levels in exosome from preadipocytes (Figure 2E). Adipocyte exosomes stained with PKH26 were added to the medium of SW480 and HCT116 recipient cells, transported into the cytoplasm of these cells, and diffused as small spots around the cell nucleus (Figure 2F).

### 2.4 Adipocyte‐Secreted Exosomes Reverse Erastin‐Induced Ferroptosis in CRC Cells

**Figure 3:**
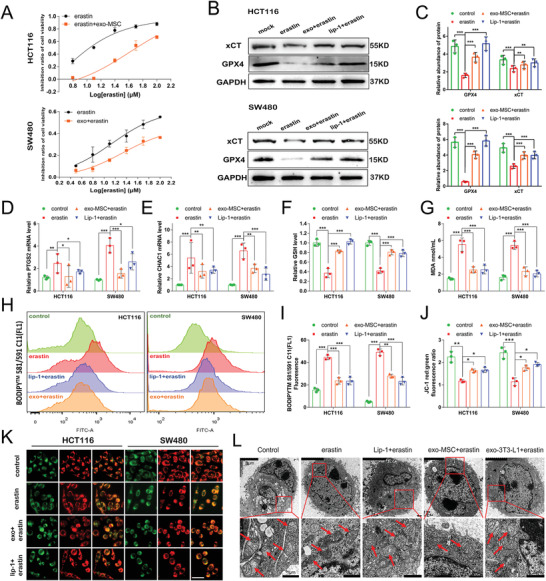
Adipocyte‐secreted exosomes reverse erastin‐induced ferroptosis in CRC cells: A) CCK‐8 detection of the inhibition ratio of erastin‐exposed CRC cells treated with or without mature adipocyte‐secreted exosomes (*n* = 3). B,C) Effects of mature adipocyte exosomes on GPX4 and xCT protein levels in CRC cells treated with erastin (B). Levels of the GPX4 and xCT proteins were determined using WB assay and quantified using gray scale analysis (C). Relative levels of PTGS2 D) and CHAC1 E) mRNA expression in CRC cells treated with mature adipocyte exosomes or lip‐1 following treatment with erastin for 24 h. Relative levels of GSH F), MDA G), C11‐BODIPY H,I) and JC‐1 red: green fluorescence ratio J,K) in control, lip‐1 or mature adipocyte exosomes following treatment with erastin for 24 h. Scale bars in (K) = 20 µm. L) Representative TEM images (scale bars: upper panel, 5 µm; lower panel, 1 µm) of mitochondrial morphology and quantification of mitochondrial damage in SW480 cells treated with lip‐1 or mature adipocyte exosomes following treatment with erastin. Red arrows indicate the mitochondria. Data are presented as means ± SD of three simultaneously performed experiments (C to G, I and J). *P* value was calculated using one‐way ANOVA; *P < 0.05, **P < 0.01, ***P < 0.001.

Different concentrations of erastin were added to SW480 and HCT116 CRC cells cocultured with adipocyte exosomes, cell viability was tested using a Cell Counting Kit‐8 (CCK‐8) after 48 h of incubation, and the inhibition ratio was calculated to explore the functions of these exosomes. Adipocyte exosomes attenuated the response of both cell lines to erastin. For HCT116 cell, IC50 value was 3.563 × 10^−6^ m in erastin group and 41.05 × 10^−6^ m in adipocyte exosome group, for SW480 cell, IC50 value was 13.84 × 10^−6^ m in erastin group and 21.53 × 10^−6^ m in adipocyte exosome group (**Figure** **3**A and Figure S3A, Supporting Information). We then examined the expression levels of the key components involved in ferroptosis pathways. As shown in Figure 3B,C and Figure S3B–E (Supporting Information), addition of erastin significantly reduced GPX4 and xCT expression, and this effect was abolished by liproxstatin‐1 treatment, consistent with the role of lip‐1, adipocyte exosomes reversed GPX4 and xCT inhibition. Ferroptosis induction is associated with the increased expression of ferroptosis marker genes such as PTGS2^[^ ^11^ ^]^ and CHAC1.^[^ ^45^ ^]^ The qPCR analysis showed significantly increased PTGS2 and CHAC1 expression levels after treatment with erastin and attenuated levels in the groups treated with adipocyte exosomes and liproxstatin‐1 (Figure 3D,E and Figure S3F,G, Supporting Information). Consistent with this findings, adipocyte exosomes decreased the formation of MDA (malondialdehyde), the end product of lipid peroxidation (Figure 3G and Figure S3J, Supporting Information). GSH consumption directly activates lipoxygenase and suppresses GPX4 activity, triggering lipid peroxidation.^[^ ^46^, ^47^ ^]^ GSH levels were significantly decreased by erastin treatment and were reversed by adipocyte exosomes (Figure 3F and Figure S3M, Supporting Information). A typical hallmark of ferroptosis is the accumulation of lipid peroxidation.^[^ ^11^ ^]^ Lipid peroxidation induced by erastin was substantially decreased upon adipocyte exosome and lip‐1 treatment (Figure 3H,I and Figure S3H,I, Supporting Information). The ratio of red to green fluorescence in JC‐1 dye‐treated cells reflected the changes in the mitochondrial membrane potential. High concentrations of JC‐1 aggregate normal mitochondria emit red fluorescence, which shifts to green fluorescence at low concentrations. After 24 h of erastin treatment, the red/green fluorescence ratio decreased, indicating a reduction in the mitochondrial membrane potential. However, the reduction was partially reversed in the adipocyte exosome pretreatment group (Figure 3J,K and Figure S3K,L, Supporting Information). Morphological changes in mitochondria are one of the typical characteristics of ferroptosis.^[^ ^48^ ^]^ Images from transmission electron microscopy show the morphology of mitochondria (red arrows) in CRC cells. Mitochondria showed an increased membrane density and a shrunken morphology by erastin stimulation, which was alleviated by addition of adipocyte exosomes (Figure 3L). These data suggest that adipose‐secreted exosomes play a pivotal role in inhibiting ferroptosis in colorectal cancer.

### 2.5 Adipose‐Derived Exosomes Transport MTTP to Inhibit Ferroptosis in CRC Cells

**Figure 4:**
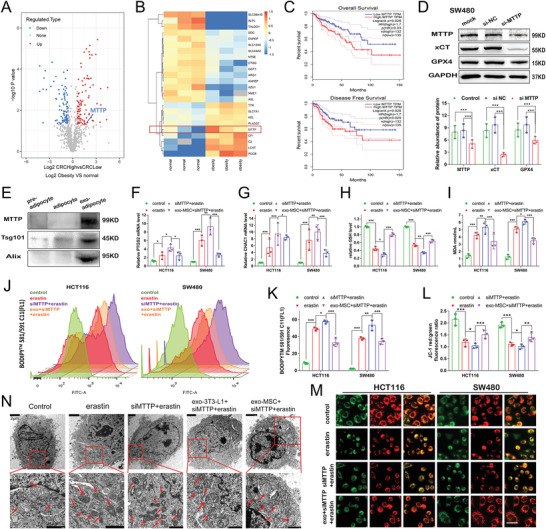
Inhibition of MTTP promotes ferroptosis in colorectal cancer cells: A) Volcano plot of exosomal proteins that were differentially expressed in the plasma of normal and obese patients. Red and blue dots represent proteins with increased or decreased expression, respectively, in the plasma of obese patients compared with normal weight patients along with the fold change. B) Heatmap of metabolism‐related proteins that were differentially expressed in plasma exosomes from normal and obese patients. C) The overall survival and disease‐free survival curves of patients with CRC stratified based on MTTP expression in the GEPIA dataset. Means and SD are shown (num (high) = 132, num (low) = 135, *P < 0.05 as determined using *t* tests). D) WB revealed the expression levels of MTTP, xCT, and GPX4 in SW480 with MTTP knockdown and quantified using a gray scale analysis. E) Quantification of the expression levels of MTTP and two representative exosome‐specific markers, Tsg101 and Alix in preadipocyte, adipocyte and mature adipocyte exosomes. Relative mRNA expression levels of PTGS2 F) and CHAC1 G) in CRC cells with MTTP knockdown or treated with mature adipocyte exosomes following treatment with erastin for 24 h. Relative levels of GSH H), MDA I), C11‐BODIPY J,K) and JC‐1 red: green fluorescence ratio L,M) in control, MTTP knockdown or mature adipocyte exosome‐treated cells following treatment with erastin for 24 h. Scale bars in (M) = 20 µm. N) Representative TEM images (scale bars: upper panel, 2 µm; lower panel, 1 µm) of mitochondrial morphology and quantification of mitochondrial damage in SW480 cells with MTTP knockdown or treated with mature adipocyte exosomes following treatment with erastin. Red arrows indicate the mitochondria. Data are presented as means ± SD of three simultaneously performed experiments (D, F to I, K, and L). *P* value was calculated using one‐way ANOVA; *P < 0.05, **P < 0.01, ***P < 0.001.

Mass spectrometry (MS) analysis was performed to compare the differentially expressed proteins in blood plasma from obese patients and patients with normal weights, as described in Figure 1F. A panel of proteins was significantly upregulated in patients with a high body fat content. A volcano plot was constructed to identify the significantly differentially expressed proteins with a middle‐high expression threshold to further explore the potential molecular mechanisms (**Figure** **4**A). Of the numerous proteins that were identified using mass spectrometry, the GO enrichment analysis revealed that proteins related to metabolic processes accounted for a substantial proportion (Figure S1D, Supporting Information). Then, we mainly focused on metabolism to simplify the interpretation of our results, conducted a heatmap analysis of metabolism‐related proteins with significantly different expression levels, and identified MTTP (Figure 4B), which was discovered as an intracellular lipid transfer protein.^[^ ^50^ ^]^ The Kaplan–Meier survival curves and log rank test showed that high MTTP expression significantly correlated with shorter overall survival (OS) and disease‐free survival (DFS) of patients with colorectal cancer (Figure 4C), suggesting that MTTP represents a factor that promotes tumor progression in CRC. Thus, we propose that MTTP may be a potential protective factor against ferroptosis. Consistent with the mass spectrometry results, western blot detection indicated that exosomes secreted by adipocytes contained MTTP (Figure 4E). MTTP knockdown in SW480 and HCT116 cells showed the downregulation of GPX4 and xCT expression (Figure 4D and Figure S4A, Supporting Information). The expression of the ferroptosis marker genes PTGS2 and CHAC1 was increased by MTTP knockdown (Figure 4F,G and Figure S4B,C, Supporting Information). Consistent with this findings, GSH levels decreased significantly after MTTP deficiency (Figure 4H and Figure S4D, Supporting Information). These results were further confirmed by analyses the accumulation of lipid peroxidation (Figure 4J,K and Figure S4F,G, Supporting Information), MDA levels (Figure 4I and Figure S4E, Supporting Information) and the mitochondrial membrane potential (Figure 4L,M and Figure S4H,I, Supporting Information). Finally, transmission electron microscopy (TEM) revealed that after erastin treatment, the typical morphological characteristics of ferroptosis, such as shrunken mitochondria and an increased membrane density, were more obvious in siMTTP‐pretreated group than in adipose exosome‐pretreated group (Figure 4N). We conclude that exosomes carrying MTTP secreted by adipose tissue reduced the level of ferroptosis in CRC cells.

### 2.6 The MTTP/PRAP1/ZEB1 Axis Suppresses Ferroptosis

**Figure 5:**
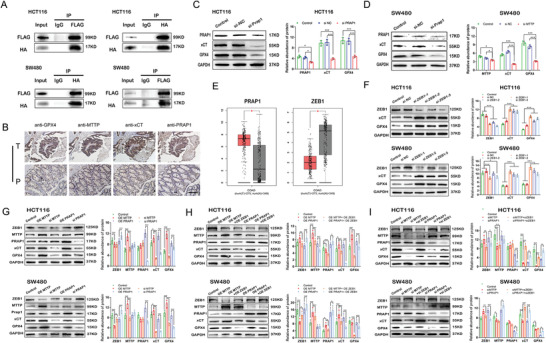
The MTTP/PRAP1/ZEB1 axis inhibits ferroptosis: A) Cell lysates from HCT‐116/SW480 cells were immunoprecipitated (IP) with an HA antibody (Ab), FLAG Ab or IgG and then subjected to western blot analysis with HA Ab or FLAG Ab. The lanes labeled Input indicate the cell lysate (*n* = 3). B) IHC analysis of GPX4, MTTP, xCT and PRAP1 levels in paired paracarcinoma tissues and tumor tissues from patients with CRC (*n* = 20). Scale bar = 200 µm. C,D) WB revealed the expression levels of PRAP1, xCT and GPX4 in HCT116 C) and SW480 D) with PRAP1 knockdown and quantified using a gray scale analysis. E) Levels of the PRAP1 transcript were significantly higher and ZEB1 mRNA levels were significantly lower in colorectal cancer tissues than in normal tissues in the GEPIA dataset. Means and SD are shown (num (T) = 275, num (P) = 349, *P < 0.05 as determined using *t* tests). F) WB detection of ZEB1, xCT and GPX4 levels in HCT116 and SW480 cells transfected with ZEB1 siRNAs and quantified by gray scale analysis. G) WB detection of ZEB1, MTTP, Prap1, xCT, and GPX4 levels in HCT116 and SW480 cells transfected with the MTTP plasmid, MTTP siRNA, Prap1 plasmid, or Prap1 siRNA and quantified by gray scale analysis. H) WB detection of ZEB1, MTTP, PRAP1, xCT, and GPX4 levels in HCT116 and SW480 cells transfected with the MTTP or PRAP1 plasmid alone or in combination with or without the ZEB1 plasmid and quantified by gray scale analysis. I) WB detection of ZEB1, MTTP, PRAP1, xCT, and GPX4 levels in HCT116 and SW480 cells transfected with the MTTP siRNA or PRAP1 siRNA in combination with or without the ZEB1 siRNA and quantified by gray scale analysis. Data are presented as means ± SD of three simultaneously performed experiments (C, D and F to I). *P* value was calculated using one‐way ANOVA; *P < 0.05, **P < 0.01, ***P < 0.001.

Coimmunoprecipitation assays revealed that Flag‐tagged MTTP was capable of pulling down HA‐tagged PRAP1 after cotransfection into CRC cells (**Figure** **5**A). Immunohistochemistry (IHC) analysis of serial sections showed a positive relationship between MTTP and PRAP1 in CRC patients (Figure 5B). PRAP1 functions to assemble proteins and ultimately regulate the cellular response to oxidative stress, and its disruption renders cancer cells more susceptible to chemotherapy.^[^ ^32^ ^]^ PRAP1 knockdown in SW480 and HCT116 cells downregulated the expression of two key ferroptosis regulators, GPX4 and xCT (Figure 5C,D). TCGA and GTEx data indicated high PRAP1 expression and low ZEB1 expression in colorectal cancer (Figure 5E). The EMT regulator^[^ ^50^ ^]^ and lipogenic factor ZEB1^[^ ^51^ ^]^ is strongly correlated with sensitivity to GPX4 inhibition and stimulated ROS production.^[^ ^34^, ^52^, ^53^ ^]^ ZEB1 inhibition in CRC cells result in upregulated GPX4 and xCT (Figure 5F). Next, the MTTP/PRAP1/ZEB1 axis was further verified. Overexpression of MTTP and PRAP1 inhibited ZEB1 expression, while knockdown of MTTP and PRAP1 promoted ZEB1 expression (Figure 5G). In the rescue experiments, when overexpression of MTTP or PRAP1 was accompanied by overexpression of ZEB1, the downregulation of ZEB1 expression was attenuated (Figure 5H). The opposite trend was observed after MTTP and PRAP1 inhibition accompanied by ZEB1 inhibition (Figure 5I). Based on these results, MTTP and PRAP1 negatively regulate ZEB1, thereby enhancing the effect of GPX4 on reducing ferroptosis susceptibility. Collectively, these results indicate that MTTP protects CRC cells from ferroptosis mainly through the MTTP/PRAP1/ZEB1 axis.

### 2.7 Adipocyte Exosomes Reduce the Sensitivity of CRC Cells to L‐OHP

**Figure 6:**
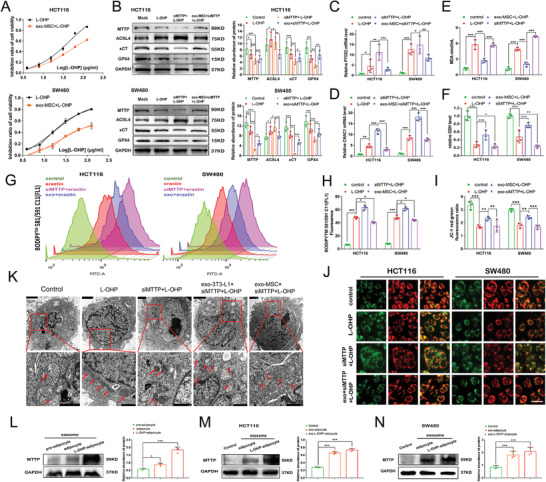
Adipocyte exosomes reduce the sensitivity of CRC cells to L‐OHP (B): A) CCK‐8 detection of the inhibition ratio of L‐OHP in CRC cells treated with or without mature adipocyte exosomes (*n* = 3). B) Effects of the MTTP siRNA and mature adipocyte exosomes on MTTP, ACSL4, xCT, and GPX4 protein levels in CRC cells treated with L‐OHP and quantified by gray scale analysis. Relative levels of PTGS2 C) and CHAC1 D) mRNA expression in CRC cells treated with mature adipocyte exosomes or the MTTP siRNA following treatment with L‐OHP were examined. Relative levels of MDA E), GSH F), C11‐BODIPY G,H) and JC‐1 red: green fluorescence ratio I,J) in control, MTTP siRNA or mature adipocyte exosome‐treated cells following treatment with L‐OHP. Scale bar in (J) = 20 µm. K) Representative TEM images (scale bars: upper panel, 2 µm; lower panel, 1 µm) of mitochondrial morphology and quantification of mitochondrial damage in SW480 cells with MTTP knockdown or treated with mature adipocyte exosomes following treatment with erastin. Red arrows indicate the mitochondria. The experiment was repeated three times independently, with similar results. L) WB detection of MTTP levels in exosomes from preadipocytes, adipocytes and adipocytes treated with L‐OHP and quantified using a gray scale analysis (*n* = 3). M,N) WB detection of MTTP levels in HCT116 and SW480 cells treated with adipocyte exosomes and L‐OHP‐adipocyte exosomes and quantified using a gray scale analysis. Data are presented as means ± SD of three simultaneously performed experiments (B to F, H, I and L to N). *P* value was calculated using one‐way ANOVA; *P < 0.05, **P < 0.01, ***P < 0.001.

The aforementioned studies raise the question of whether ferroptosis inhibition might enhance resistance to chemotherapy in patients with CRC. SW480 and HCT116 cells were cocultured with adipocyte exosomes and treated with different concentrations of L‐OHP. After 48 h, Cell Counting Kit‐8 (CCK‐8) was used to detect cell viability and calculated the inhibition ratio. Adipocyte exosomes mitigated the response of both cell lines to L‐OHP (**Figure** **6**A and Figure S5A, Supporting Information). As shown in Figure 6B and Figure S5B, Supporting Information, the addition of L‐OHP significantly reduced MTTP, GPX4 and xCT expression while increasing ACSL4 expression. However, this effect was reversed following adipocyte exosome treatment. qPCR analysis showed significantly increased PTGS2 (Figure 6C and Figure S5C, Supporting Information) and CHAC1 (Figure 6D and Figure S5D, Supporting Information) expression levels in CRC cells transfected with MTTP siRNA and reduced levels in the adipose exosome group. Consistent with these results, levels of MDA formation (Figure 6E and Figure S5G, Supporting Information) and lipid peroxidation (Figure 6G,H and Figure S5E,F, Supporting Information) were increased by MTTP knockdown while reduced in adipose exosome group. Subsequent GSH (Figure 6F and Figure S5J, Supporting Information) and mitochondrial membrane potential assays (Figure 6I,J and Figure S5H,I, Supporting Information) further confirmed that ferroptosis susceptibility was increased by MTTP deletion while suppressed by adipose exosomes. TEM images show the morphology of mitochondria (red arrows) in SW480 cells treated with L‐OHP for 24 h. An increased membrane density and a shrunken morphology of mitochondria were observed in the MTTP knockdown group, which was alleviated by the addition of adipocyte exosomes (Figure 6K). After oxaliplatin stimulation for 7 days, MTTP expression was increased in mature adipocytes and significantly increased in exosomes secreted by adipocytes compared with preadipocytes (Figure 6L). Then, adipocyte exosomes were cocultured with CRC cells, and the results showed that oxaliplatin‐stimulated exosomes significantly increased the expression of MTTP in HCT116 (Figure 6M) and SW480 (Figure 6N). Therefore, MTTP is involved in the regulation of ferroptosis and results in acquired chemoresistance, and chemotoxicity promotes MTTP secretion and expression in adipocytes.

### 2.8 The Roles of KD‐MTTP Exosomes and Adipocyte Exosomes were Verified in CRC Organoids

**Figure 7:**
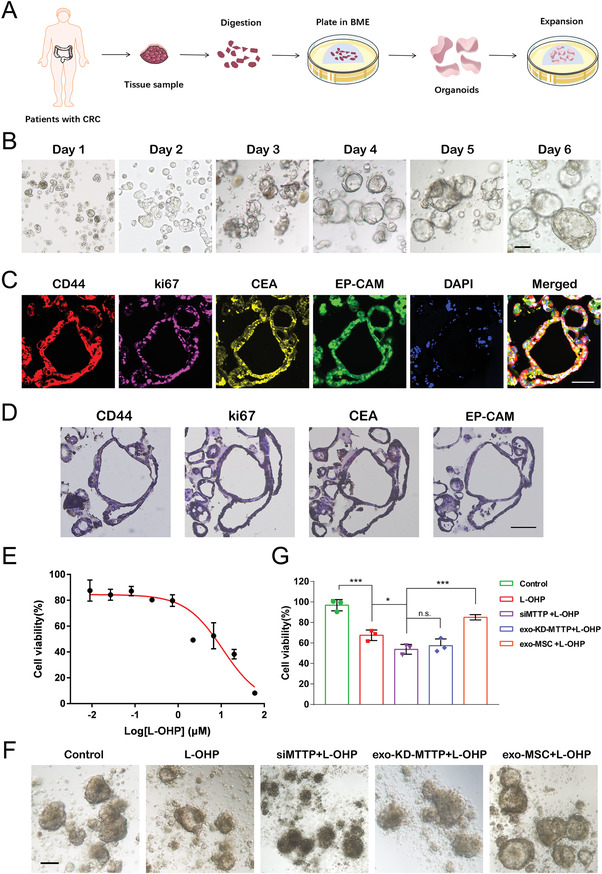
Adipocyte exosomes reduce the sensitivity of CRC organoids to L‐OHP: A) Schematic description of the organoids development. B) Representative images of organoids growth were recorded by bright‐field microscope. Scale bar = 100 µm. C) Multiplex Fluorescent Immunohistochemistry Staining images of organoids expressing specific markers CD44 (red), Ki67 (pink), CEA (yellow) and EP‐CAM (green) with blue DAPI staining (scale bar = 500 µm). D) Immunohistochemistry analysis of CD44, Ki67, CEA and EP‐CAM levels in serial organoids sections (scale bar = 500 µm). E) Cell viability assay detection of the viability of oxaliplatin‐exposed organoids. F) Representative images of organoids treated with L‐OHP, pretreated with MTTP siRNA, exosomes secreted by KD‐MTTP adipocytes or mature adipocyte exosomes and then treated with oxaliplatin (scale bar = 200 µm). G) Cell viability of organoids described in (F). Data are presented as means ± SD of three simultaneously performed experiments (G). *P* value was calculated using one‐way ANOVA; n.s., not significant; *P < 0.05, **P < 0.01, ***P < 0.001.

The CRC tissue isolation and organoids culture process is shown in **Figure** **7**A. For assessment of organoid growth, images of organoids were taken every day for a week after resuscitation (Figure 7B). To further assess CRC organoids, immunohistochemical multiplex fluorescent IHC and immunohistochemical analysis showed that organoids expressed the stemness‐specific surface marker CD44, the epithelial cell adhesion molecule (Ep‐CAM) and the colorectal adenocarcinoma biomarker CEA. Finally, high Ki67 staining indicated high proliferative activity (Figure 7C,D), confirming the epithelial origin of the CRC organoids and contamination with other cell types. After organoid formation, different concentrations of oxaliplatin were added to the organoids, and the IC50 (10.84 × 10^−6^ m) was calculated after seven days of medication (Figure 7E). The organoids were divided into five groups: the control group, the oxaliplatin group, the MTTP knockdown, the adipocyte exosomes coculture and the KD‐MTTP exosomes coculture then add oxaliplatin group. The organoids were treated with 10.84 µM oxaliplatin. After 7 days of incubation, cell viability was determined by a CellTiter‐Glo Luminescent assay (Promega G7573) to explore the functions of these exosomes. As shown in Figure 7F,G, the response of organoids to oxaliplatin was enhanced by KD‐MTTP exosomes while attenuated by adipocyte exosomes.

### 2.9 Exosomal MTTP Inhibits Ferroptosis by Decreasing PUFA Levels in CRC Cells

**Figure 8:**
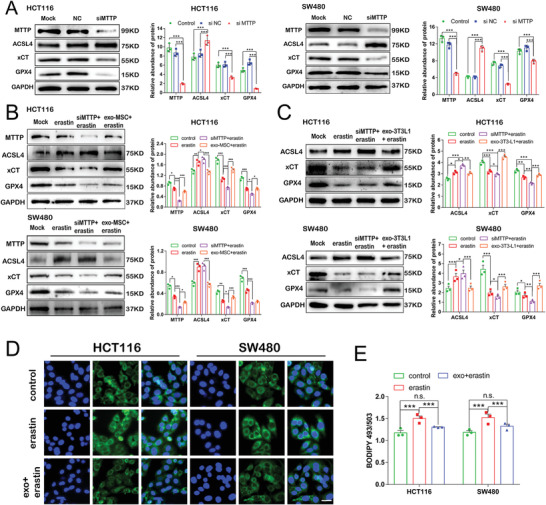
Exosomal MTTP inhibits ferroptosis by decreasing PUFA levels in CRC cells: A) WB revealed the expression levels of MTTP, ACSL4, xCT, and GPX4 in HCT116 and SW480 cells with MTTP knockdown and quantified by gray scale analysis. B,C) WB detection of ACSL4, xCT, and GPX4 levels in CRC cells with MTTP knockdown or treated with mature adipocyte exosomes secreted by MSC B) and 3T3‐L1 C) following treatment with erastin for 24 h. Then quantified by gray scale analysis. (D, E) CRC cells treated with erastin or adipocyte exosomes and erastin pretreated with arachidonic acid (AA) for 24 h (scale bar = 20 µm). Data are presented as means ± SD of three simultaneously performed experiments (A to C, E). *P* value was calculated using one‐way ANOVA; n.s., not significant; *P < 0.05, **P < 0.01, ***P < 0.001.

MTTP plays a role in promoting fatty acid transport. We found that ACSL4 expression was significantly increased by MTTP deletion in HCT116 and SW480 cells (**Figure** **8**A) and was reversed by adipose exosome pretreatment (Figure 8B,C). SW480 and HCT116 cells were treated with AA for 24 h and stained with BODIPY‐493/503 dyes to detect the fatty acid uptake ratio and explore the mechanisms. Green signals in the cytoplasm suggested that adipocyte exosomes inhibited AA uptake in CRC cells (Figure 8D,E). Based on these results, adipocyte exosomes provide MTTP and inhibit ferroptosis by inhibiting fatty acid uptake. Together, our data suggest that exosomes secreted from adipose tissue reduce PUFA levels by downregulating ACSL4 and upregulating xCT expression.

### 2.10 Exosomal MTTP in Promotes Oxaliplatin Resistance in Vivo by Suppressing Ferroptosis

**Figure 9:**
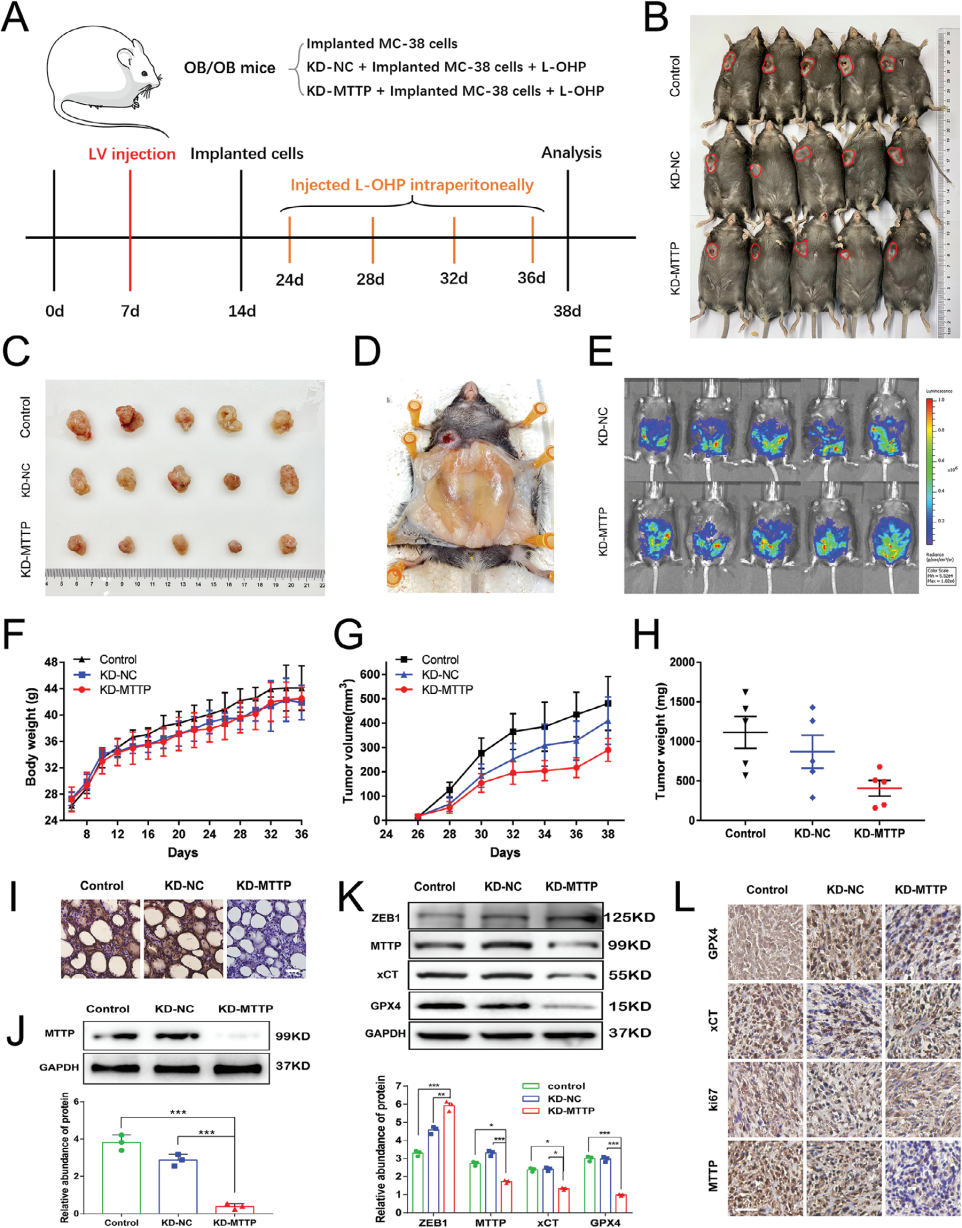
Effects of adipocyte exosomes on colorectal carcinoma in a murine model: A) Schematic description of the experimental design for the in vivo models. B,C) Photos of mice and tumors from all groups. D) Anatomical location of abdominal fat pad in ob/ob mouce. E) In vivo fluorescence signals were recorded by IVIS SPECTRUM. Fluorescence was detected at the abdominal fat pad. Mouse weights F), tumor volumes G) and tumor weights H) in each group (*n* = 5). I) IHC analysis of MTTP levels in abdominal fat pad in each group (*n* = 5). Scale bar = 50 µm. J) WB analysis of MTTP protein levels in abdominal fat pad in each group and quantified using a gray scale analysis. K) Expression of ZEB1, MTTP, GPX4 and xCT detected by western blot and quantified using a gray scale analysis (*n* = 5). L) IHC analysis of GPX4, xCT, ki67, and MTTP levels of tumors in each group (*n* = 5). Scale bar = 50 µm. Data are presented as means ± SD of three simultaneously performed experiments J,K). *P* value was calculated using one‐way ANOVA; *P < 0.05, **P < 0.01, ***P < 0.001.

**Figure 10:**
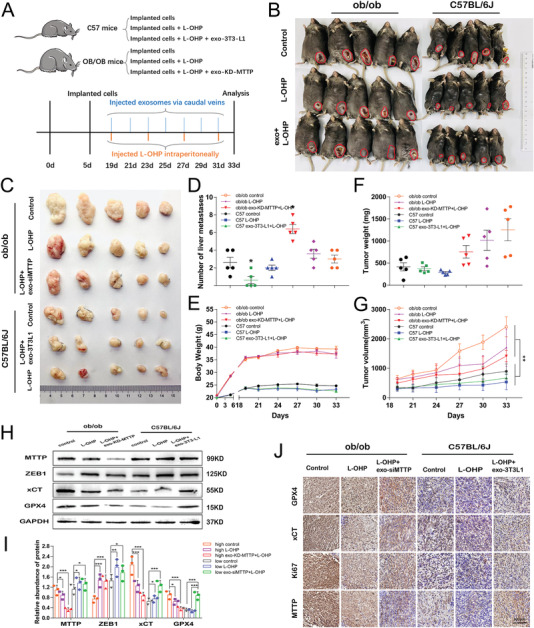
Effects of adipocyte exosomes on colorectal carcinoma in a murine model: A) Schematic description of the experimental design for the in vivo models. B,C) Photos of mice and tumors from all groups. D) Numbers of liver metastases in each group (*n* = 5). Mouse weights E), tumor weights F), and tumor volumes G) in each group (*n* = 5). WB analysis of ZEB1, MTTP, GPX4, and xCT protein levels in tumors H) and quantified by a gray scale analysis I). J) Expression of MTTP, GPX4, xCT, and ki67 detected using IHC (*n* = 5). Data are presented as means ± SD of three simultaneously performed experiments (G,I). *P* value was calculated using one‐way ANOVA; *P < 0.05, **P < 0.01, ***P < 0.001.

**Figure 11:**
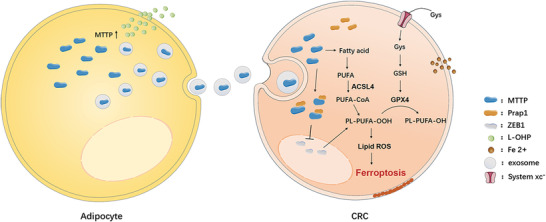
A proposed model illustrating the role of adipose‐derived exosomal MTTP in regulating ferroptosis in CRC cells.

The effects of exosomes secreted from adipocytes on tumor growth in vivo were studied using tumor‐implanted mice. ob/ob mice are considered an optimal model to simulate an obese condition. Thus, we chose them as obesity models to explore the role of adipocyte exosomes in reversing oxaliplatin resistance in vivo. As shown in **Figure** **9**A, each group contained 5 ob/ob mice. Subcutaneous implantation of colorectal tumor cells (SW480) was performed. On day 10 after tumor implantation, 8 mg/kg L‐OHP was injected intraperitoneally every four days. On day 38, all mice were sacrificed for analysis. The major and minor axes of tumors were measured every 3 days, and the tumor volumes were calculated. All mice were euthanized and photographed (Figure 9B). Photos of tumors obtained from each group are shown in Figure 9C. The anatomical location of abdominal fat pad in ob/ob mouce was shown in Figure 9D. The infection efficiency lentivirus on abdominal fat pads was monitored on the 14th day after surgery via an IVIS SPECTRUM in vivo optical imaging system. Figure 9E shows that the abdominal fat pads were successfully infected. During the tumor‐bearing stage, the body weight (Figure 9F) and tumor growth (Figure 9G) of the mice were recorded. In general, the tumor volumes (Figure 9G) and tumor weights (Figure 9H) of the KD‐MTTP group were noticeably smaller than those of the other two groups. The levels of MTTP expression in lentivirus‐infected abdominal fat pads were significantly decreased (Figure 9I,J). Changes in the protein content in transplanted tumor tissues were detected, and the expression of GPX4, xCT and MTTP was decreased in the KD‐MTTP group (Figure 9K). Immunohistochemical staining showed relative expression of GPX4, xCT, MTTP and ki67 in tumor tissues from ob/ob mice. The expression level of MTTP in the KD‐MTTP group was significantly decreased, and GPX4 and xCT levels in the KD‐MTTP group were also slightly decreased (Figure 9L). Consistent with our in vitro observations, this finding suggest that adipose‐derived exosomes deliver MTTP to tumors, resulting in reduced sensitivity to oxaliplatin in vivo. Then, exogenous exosomes‐KD‐MTTP were used, as shown in **Figure** **10**A, each group contained 5 mice. Subcutaneous implantation of colorectal tumor cells (SW480) was performed in ob/ob mice and C57 mice simultaneously. On Day 14 after tumor implantation, 50 µg of exosomes were injected caudally every other day and 8 mg kg L‐OHP was injected intraperitoneally every four days. On Day 33, all mice were sacrificed for analysis. The major and minor axes of tumors were measured every 3 days, and the tumor volumes were calculated. All mice were euthanized and photographed (Figure 10B). Photos of tumors obtained from each group are shown in Figure 10C. The tumor volumes of ob/ob mice were noticeably larger than those of C57 mice in general. The number of liver metastases (Figure 10D) in C57 mice was significantly lower than that in ob/ob mice, while this phenomenon was reversed by the injection of adipocyte exosomes. During the tumor‐bearing stage, the body weight (Figure 10E) and tumor growth (Figure 10F) of mice were recorded. The weight of the ob/ob mice ranged from 35 to 40 g, and C57 mice weighed ≈22 g. The largest volume of tumors (Figure 10G) was observed in the control group of ob/ob mice and lowest in the L‐OHP group of C57 mice. Changes in the protein content in transplanted tumor tissues were detected, and the expression of GPX4, xCT and MTTP was decreased in the L‐OHP injection group, which was reversed by treatment with adipocyte exosomes (Figure 10H,I). Immunohistochemical staining showed relatively expression of GPX4, xCT, MTTP and ki67 in tumor tissues from ob/ob mice than in those from C57 mice, and these changes were inhibited by treatment with L‐OHP and recovered by injecting adipocyte exosomes (Figure 10J). Consistent with our in vitro observations, this finding suggested that adipose‐derived exosomes deliver MTTP to tumors, resulting in reduced sensitivity to oxaliplatin in vivo (**Figure** **11**).

## 3 Discussion

Obesity is associated with the development of multiple cancers, including liver cancer, breast cancer, kidney cancer, bladder cancer and endometrial cancer,^[^ ^54^ ^]^ and has been identified as a well‐established risk factor for cancer and cancer‐related mortality.^[^ ^37^ ^]^ It was reported that obesity is closely related to T‐cell dysfunction in many types of cancer.^[^ ^55^ ^]^ Adipocyte‐derived inflammatory cytokines, including IL‐8 and IL‐6, promote epithelial‐mesenchymal transition, metastasis and tumor progression.^[^ ^56^ ^]^ A worse prognosis and higher recurrence rates of cancer after chemotherapy are more frequently observed in obese patients.^[^ ^57^ ^]^ Consequently, studies aiming to elucidate the molecular mechanisms underlying the link between obesity and chemoresistance in CRC are urgently needed.

Ferroptosis is characterized by the accumulation of lipid peroxidation products and lethal ROS.^[^ ^11^, ^12^ ^]^ The differences in the intracellular levels of iron and GSH between cancerous and normal cells may be exploited for targeted therapy based on ferroptosis.^[^ ^58^, ^59^ ^]^ Emerging evidence has shown the potential of ferroptosis in cancer therapy, particularly in aggressive malignancies that are resistant to conventional therapies.^[^ ^60^, ^61^ ^]^ ACSL4 affects the sensitivity to ferroptosis by altering cellular lipid composition.^[^ ^25^ ^]^ Moreover, inhibition of ACSL4 expression in CRC cells protects against cell damage by attenuating ferroptosis.^[^ ^62^ ^]^ Sulfasalazine (SSZ), a drug targeting the cystine transporter, can reverse platinum resistance in CRC through a GSH‐dependent mechanism.^[^ ^63^ ^]^ Overexpression of SLC7A11 in tumors inhibits ROS‐induced ferroptosis and eliminates p53‐mediated inhibition of tumor growth.^[^ ^64^ ^]^ Furthermore, CD44v interacts with xCT and stabilizes xCT, thereby promoting cysteine uptake in CRC cells to synthesize GSH, thus attenuating chemotherapy resistance in CRC cells.^[^ ^65^ ^]^ Neutrophil infiltration leads to ferroptotic death of tumor cells.^[^ ^66^ ^]^ CD8^+^ T‐cell‐derived IFN*γ* cooperates with radiotherapy and inhibits SLC7A11 expression to promote lipid oxidation in tumor cells.^[^ ^67^ ^]^ RT‐MPs render tumor cells more vulnerable to macrophages mainly by inducing ferroptosis in immunogenic cells.^[^ ^68^ ^]^ However, the potential roles of adipose‐secreted exosomes in regulating ferroptosis of tumor cells are still unknown.

Chemotherapy is the main treatment for patients with advanced CRC, and the oxaliplatin‐based XELOX and FOLFOX regimens are the recommended first‐line chemotherapy regimens. However, the rapid and common development of oxaliplatin resistance limits CRC therapeutic efficacy.^[^ ^69^ ^]^ Hence, other methods that efficiently prohibit the growth of CRC cells must be identified. Chemotherapeutic resistance is commonly associated with DNA damage repair, molecular mutations that regulate cell apoptosis, and elevated glutathione (GSH) concentrations.^[^ ^70^ ^]^ Changes in ferroptosis‐related signaling pathways may represent an approach to subvert chemotherapy resistance. Therapy‐resistant cancer cells are sensitive to treatments targeting the lipid peroxidase pathway.^[^ ^34^ ^]^ Inhibition of the p62‐Keap1‐NRF2 pathway reverses sorafenib resistance in HCC cells.^[^ ^71^ ^]^ Hence, exploring the role of ferroptosis in chemotherapy resistance in CRC is imperative. Exosomes promote the development of chemotherapy resistance in tumor cells through various mechanisms.^[^ ^72^ ^]^ It has been reported that exosomes secreted by cancer‐associated fibroblasts (CAFs) promote chemotherapy resistance in gastric cancer by inhibiting ferroptosis.^[^ ^13^ ^]^ Our study showed that adipose tissue‐secreted exosomes inhibit lipid ROS production and reduce ferroptosis susceptibility, thus reversing chemotherapy resistance in CRC cells.

In the current study, clinical characteristics of patients with advanced CRC treated in our hospital showed that a high body fat ratio was associated with cancer progression and a poor prognosis. Adipocyte exosomes reduced the levels of MDA and lipid peroxidation, which suppressed ferroptosis in CRC cells. MTTP, an intracellular lipid transfer protein, was upregulated in the plasma exosomes of obese patients and played a leading role in suppressing lipid peroxidase activity. We injected KD‐MTTP lentivirus into the abdominal fat pads of obese mice and conducted experiments in CRC organoids to further validate the MTTP/PRAP1/ZEB1 signaling pathway, which may provide important insights for reversing chemotherapy resistance. Currently, new treatment options, including targeted therapy and immunotherapy, are increasingly being applied to personalize treatment in the clinic. Further studies are needed to assess treatment resistance and ferroptosis in obese patients and achieve better treatment outcomes.

## 4 Experimental Section

### Human Tissues

All clinical tumor tissue samples and plasma samples were obtained from patients who were histopathologically and clinically diagnosed with advanced CRC and obtained from Tianjin Medical University Cancer Institute and Hospital. The tumors were fixed with 4% paraformaldehyde, embedded in paraffin and then stained with antibodies. The study methodologies conformed with standards of the Declaration of Helsinki, and all aspects of this study were approved by the Ethics Committee of Tianjin Medical University Cancer Institute and Hospital (EK2019039), the informed consent was obtained from all patients.

### Animals

Male ob/ob mice were purchased from GemPharmatech (Jiangsu, China), arrived at 4 weeks of age and housed in a special pathogen‐free animal facility with a controlled environment (12 h light/dark cycle, 21 ± 2 °C, humidity 50 ± 10%). Mice were randomized into 3 groups according to the baseline body weight and were provided ad libitum access to tap water and chow. All procedures were performed in accordance with The Institutional Animal Care and Research Advisory Committee of Tianjin Medical University Cancer Institute and Hospital (LLSP2019‐013).

### Cell Culture

SW480 and HCT116 (human colorectal cancer cells), MC38 (mouse colorectal cancer cells) and 3T3‐L1 (murine white preadipocytes) were obtained from the Cell Bank of the Chinese Academy of Sciences (Shanghai, China). SW480, HCT116 and MC38 cells were cultured in RPMI 1640 medium (Gibco, Grand Island, NY, USA). 3T3‐L1 cells were cultured in DMEM/F12 (Gibco, Grand Island, NY, USA). All cells were cultured with 10% fetal bovine serum (Gibco, NY, USA) and 1% penicillin/streptomycin (Gibco, NY, USA) at 37 °C with 5% CO_2_ in a humidified incubator (Thermo, USA). Mycoplasma contamination was detected in each cell line before use.

### Plasmid Construction and Cell Transfection

MTTP‐siRNA, PRAP1‐siRNA, and ZEB1‐siRNA (RiboBio, Guangzhou) and Flag‐MTTP, HA‐PRAP1 and ZEB1 plasmids (Hanbio Biotechnology, Shanghai) were transfected using Lipofectamine 2000 (Invitrogen) according to the manufacturer's instructions. The transfection efficiency was measured using western blotting.

### Adipocyte Differentiation

MSC and 3T3‐L1 cells were cultured in DMEM/F12 (Gibco, Beijing, China). Cells were induced with 0.5 × 10^−3^ m 3‐isobutyl‐1‐methylxanthine (IBMX) (Sigma, MA, USA), 10 µg mL^−1^ insulin (Novolin R), 2.5 × 10^−6^ m dexamethasone (DEX, Sigma, MA, USA) and 125 × 10^−3^ m indomethacin (Sigma, MA, USA). Forty‐eight hours later, fresh medium containing 10 µg mL^−1^ insulin was applied. Fresh standard DMEM/F12 was applied every other day until cells were differentiated at Day 10.

### Oil Red O Staining

MSC and 3T3‐L1 preadipocytes were seeded in a 12‐well plate and allowed to reach 70% confluence. Then, cells were cultured with differentiation media for 10 days. The cells were washed with phosphate‐buffered saline (PBS), fixed with 10% formalin for 15 min at room temperature, and then washed again with deionized water three times. The Oil Red O working solution (Beyotime, China) was added to the cells, incubated for 30 min at 37 °C, and then washed three times with deionized water.

### Isolation of Exosomes from Cell Culture Medium and Plasma

Complete FBS was ultracentrifuged at 100 000 × *g* (rotor: SW 32 Ti, Beckman Coulter, CA, USA) for 16 h to obtain exosome‐free FBS. When adipocytes were differentiated, the medium was collected and centrifuged at 3000 × *g* for 15 min to remove cell debris. Then, the supernatant was centrifuged at 10 000 × *g* for 15 min to discard large vesicles. Eventually, the supernatant was collected and ultracentrifuged at 100 000 × *g* for 90 min, and all steps were performed at 4 °C. Exosomes were resuspended in PBS and filtered to remove bacteria with a 0.22 × 10^−6^ m filter (Millex, 2513601HA, USA). The concentration of exosomes was quantified using a NanoDrop 2000 Spectrophotometer (Thermo, Waltham, MA, USA).

### Transmission Electron Microscopy (TEM) Imaging

For imaging mitochondria, CRC cells were digested with 0.05% trypsin, collected in a 1.5 mL microcentrifuge tube and immediately centrifuged at 1000 rpm min^−1^ for 5 min to remove trypsin. Then, human serum was added and mixed before resuspension. The cell mass was fixed with 2.5% glutaraldehyde diluted in phosphate buffer, stored overnight at 4 °C, and stained with 1% OsO_4_. All samples were dehydrated in a gradient of alcohol solutions and sectioned into ultrathin sections. For imaging exosome, the exosome pellet was placed in a droplet of 2.5% glutaraldehyde in PBS buffer at pH 7.2 and fixed overnight at 4 °C. Samples were rinsed with PBS (3 times, 10 min each), postfixed with 1% osmium tetroxide for 60 min, embedded in 10% gelatin, fixed with glutaraldehyde at 4 °C, and cut into several blocks (less than 1 mm^3^). The samples were dehydrated with increasing concentrations of alcohol (30, 50, 70, 90, 95, and 100% × 3). Pure alcohol was exchanged with propylene oxide, and specimens were infiltrated with increasing concentrations (25, 50, 75, and 100%) of Quetol‐812 epoxy resin mixed with propylene oxide for a minimum of 3 h per step. Samples were embedded in pure, fresh Quetol‐812 epoxy resin and polymerized at 35 °C for 12 h, 45 °C for 12 h, and 60 °C for 24 h. Ultrathin sections (100 nm) were cut using a Leica UC6 ultramicrotome and poststained with uranyl acetate for 10 min and lead citrate for 5 min before observation using an FEI Tecnai T20 transmission electron microscope (Thermo, Waltham, MA, USA) operated at 120 kV.

### Nanoparticle Tracking Analysis (NTA)

The size and density of exosomes were tracked using the Nanosight NS 300 system (NanoSight Technology, Malvern, UK). Exosomes were resuspended in PBS and further diluted to achieve between 20 and 100 objects per frame. The samples were injected into the sample chamber at ambient temperature. Samples were imaged with a 488 nm laser and a high‐sensitivity sCMOS camera and were measured in triplicate at camera setting 13 with an acquisition time of 30 s and a detection threshold setting of 7. At least 200 completed tracks were analyzed in each video. All data were analyzed using NTA analytical software (version 2.3).

### Size Distribution and Particle Concentration Measurement of Small Extracellular Vesicles (sEVs)

The size distribution and particle concentration of exosomes were measured by using a nanoflow cytometer (N30E Nanoflow Analyzer, NanoFCM Inc., Xiamen, China) at EchoBiotech Co. Ltd., Beijing, P. R. China. Briefly, the side scatter intensity (SSI) was measured by loading standard polystyrene nanoparticles (250 nm) into a nanoflow cytometer. Next, isolated exosome samples diluted with PBS (to 1–10 ng µL^−1^ according to the BCA Protein Assay results) were loaded into the nanoflow to measure the SSI. Finally, the concentration of EVs was calculated according to the ratio of SSI to particle concentration in the standard polystyrene nanoparticles. For size measurement, standard silica nanoparticles with mixed sizes (68, 91, 113, 155 nm) were loaded into the nanoflow cytometer to generate a standard curve, and the exosome sample was loaded. The size distribution was calculated according to the standard curve.

### PKH26 staining

The PKH26 Red Fluorescent Cell Linker Kit (Sigma, USA) was utilized to stain the lipid bilayer. Exosomes were resuspended in 100 µL of diluent C and then incubated with 100 µL of PKH26 dye solution for 5 min. The incubation was stopped by adding 500 µL of serum. The labeled exosomes were washed with PBS and incubated with recipient cells in a well of a 12‐well plate for 8 h before imaging.

### Protein Extraction and Western Blotting

Total protein was isolated with RIPA buffer from cultured cells and exosomes, heated at 95 °C for 10 min and quantified using a NanoDrop2000 spectrophotometer. Each sample was separated on 10% SDS–PAGE gels and transferred onto PVDF membranes (Roche, Basel, Switzerland). Then, membranes were blocked for 1 h and incubated with primary antibodies, including anti‐MTP (1:500; Santa Cruz, sc‐135994), anti‐ACSL4 (1:500; Santa Cruz, sc‐365230), anti‐PRAP1 (1:1000; Proteintech,11932‐1‐AP), anti‐xCT (1:1000; Abcam, ab175186), anti‐GPX4 (1:1000; R&D,#565320), anti‐ZEB1 (1:1000; CST; #70512), anti‐CD9 (1:1000; CST; #13403), anti‐Alix (1:1000; CST; #92880), anti‐CD69 (1:1000; CST; #28633), anti‐TSG101 (1:500; Santa Cruz, sc‐7964) and anti‐GAPDH (1:3000, Santa Cruz, sc‐32233), for at least 16 h at 4 °C. Then, the membranes were incubated with the corresponding secondary antibodies at a 1:5000 dilution for 1 h at room temperature. All samples were normalized to GAPDH.

### Coimmunoprecipitation (Co‐IP)

Cell lysates were prepared as follows: 150 × 10^−3^ m KCl, 25 × 10^−3^ m Tris–HCl, pH 7.4, 5 × 10^−3^ m EDTA, 0.5% Triton X‐100, 5 × 10^−3^ m dithiothreitol (DTT), PMSF and cocktail. The supernatant was incubated with an anti‐HA antibody (1:1000; Abmart; M20003), anti‐FLAG antibody (1:1000; Abmart; M20008) or IgG (1:3000; CST; #2729) at 4 °C overnight and then cocultured with Protein A/G beads (Santa Cruz; sc‐2003) for 2–4 h at 4 °C. The beads were washed 3 times with lysis buffer, and western blot analysis was performed.

### RNA Isolation and Quantitative RT‐PCR

Total RNA was extracted from cultured cells with TRIzol reagent (Invitrogen). Two micrograms of RNA were utilized for reverse transcription PCR (Eppendorf AG 22331 Hamburg, Germany) to synthesize cDNAs with Reverse Transcriptase M‐MLV (TaKaRa, Osaka, Japan). Then, quantitative RT‐PCR was performed using a SYBR Green mixture and a QuantStudio 5 Flex real‐time PCR system (Applied Biosystems). All samples were assayed in triplicate, and the relative mRNA level normalized to the control was calculated with the equation 2^–ΔCT^ (ΔCT = CT gene − CT control). A comparative CT method was used to compare samples with the control groups, and all mRNA levels were normalized to GAPDH. Primers for PTGS2, CHAC1 and GAPDH were as follows:

PTGS2 primers
Forward 5′‐CTGGCGCTCAGCCATACAG‐3′
Reverse 5′‐CGCACTTATACTGGTCAAATCCC‐3′
CHAC1 primers
Forward 5′‐GACGCTCCTTGAAGATCATGAG‐3′
Reverse 5′‐CAGCAAGTATTCAAGGTTGTGG‐3′
GAPDH primers
Forward 5′‐TGCACCACCAACTGCTTAGC‐3′
Reverse 5′‐GGCATGGACTGTGGTCATGAG‐3′

### Cell Viability Assay

Cell viability was assayed using a CCK‐8 kit (Solarbio, CA1210). Cells were seeded in 96‐well plates and incubated with the indicated treatments at various doses for 48 h. Subsequently, 100 µL of fresh medium with 10 µL of CCK‐8 solution were added and incubated in the dark for 2.5 h (37 °C, 5% CO_2_). The absorbance was measured at a wavelength of 450 nm using a microplate reader (Thermo). The inhibition rate was calculated using the following formula

(1) $$\begin{array}{ccc} \mathrm{Inhibition}\;\mathrm{ratio} & = & \left({\mathrm{OD}}_{\mathrm{control}}-{\mathrm{OD}}_{\mathrm{experiment}}\right)/\left({\mathrm{OD}}_{\mathrm{control}}-{\mathrm{OD}}_{\mathrm{blank}}\right)\\ & & \times \;100\% \end{array}$$

### MDA Assay

Cells were plated in 6‐well cell culture plates (Corning). The procedures were consistent with the C11‐BODIPY assay. Protein concentrations were measured using a NanoDrop 2000 spectrophotometer (Thermo, Waltham, MA, USA), and MDA levels were then detected using a lipid peroxidation MDA assay kit (Abcam, UK; ab118970) according to the instructions. The MDA content was measured, and the ratio of MDA levels to the protein concentration was calculated.

### GSH Assay

The relative GSH concentration in cell lysates was assessed using a kit (#A006‐2‐1, Nanjing Jiancheng Bioengineering Institute, China) according to the manufacturer's instructions. The reaction rate was proportional to the concentration of GSH. The absorbance of the yellow product (5‐thio‐2‐nitrobenzoic acid) was measured spectrophotometrically at 415 nm using a microplate reader. The relative level of GSH in all groups was calculated and normalized to the protein concentration.

### C11‐BODIPY Assay

Cells were plated in 6‐well plates (Corning), and the culture media was replaced with serum‐free media containing 2 µmol L^−1^ C11‐BODIPY (Thermo Fisher Scientific, D3861) for staining and cells were incubated in the dark for 30 min to determine the lipid ROS levels. Then, the cells were dissociated, washed and resuspended in 400 µl for PBS before flow cytometry (BD Biosciences) analysis. Fluorescence was determined at an excitation wavelength of 488 nm and an emission wavelength of 525 nm. The fluorescence intensity of each group indicated the amount of lipid ROS.

### Mitochondrial Membrane Potential (MMP) Detection

Cells were incubated with 500 µL of JC‐1 staining working solution for 20 min at 37 °C (C2003S, Beyotime, China). The fluorescent dye‐labeled cells were washed with PBS twice and images were captured using a fluorescence microscope (Zeiss 2.0, Germany).

### BODIPY‐493/503 Staining

Cell lines were pretreated with AA (100 × 10^−9^ m) for 24 h. Then, the supernatant was discarded and cells were stained with BODIPY‐493/503 labeling fatty acids (1 µg mL^−1^) for 30 min and DAPI (1:1000 diluted) for 10 min for nuclear staining. For confocal microscopy, a Zeiss C2 Plus confocal microscope was used.

### Immunohistochemical (IHC) Staining

Pairs of paraffin‐embedded paired tumor tissues and paracarcinoma tissues were stained with 1:1000 dilutions of anti‐xCT (Abcam, Cambridge, UK; ab175186) or anti‐GPX4 (1:1000; R&D Systems, #565320), a 1:500 dilution of the anti‐MTTP antibody (Santa; sc‐135994), anti‐PRAP1 (1: 1000; Proteintech, 11932‐1‐AP) antibodies. A DAB system (Zhongshan Jinqiao, China) was used to identify the protein expression level. The positive staining from at least five sections was quantified.

### Mass Spectrometry Analysis and Bioinformatics Analysis

The exosome samples were prepared in three biological replicates from the plasma of 45 obese patients and 30 patients with normal weight who were all diagnosed with CRC (Figure 1A, Supporting Information). Then, the samples were processed for tandem mass tag (TMT) quantitative proteomic analysis by PTM BioLab (Hangzhou, China). The peptides were exposed to an NSI source followed by tandem mass spectrometry (MS/MS) in a Q Exactive Plus mass spectrometer (Thermo Scientific) coupled online to the UPLC. The *m*/*z* scan range was 350 to 1800 for the full scan, and intact peptides were acquired in the Orbitrap at a resolution of 70 000. Peptides were then selected for MS/MS using an NCE setting of 28, and the fragments were detected in the Orbitrap at a resolution of 17 500. A procedure was performed that alternated between one MS scan followed by 20 MS/MS scans with 15.0 s dynamic exclusion. The automatic gain control (AGC) was set to 5E4. The fixed first mass was set to 100 m/z. The MS/MS data were processed using the MaxQuant search engine (v.1.5.2.8). Differential expression analyses were performed using limma (R packages v3.38.3) with a cutoff of a 1.44‐fold change and *P* value < 0.0522. KEGG pathway enrichment analysis was performed with clusterProfiler (v3.10.1)23. Plots were generated with ggplot2 (v3.2.1) and ggpubr (v0.2.4). A heatmap of upregulated genes and downregulated genes was plotted with pheatmap (v1.0.12).

### Tissue Processing and Organoid Culture

Colorectal cancer organoids were derived from surgery samples of advanced CRC patients at Tianjin Medical University Cancer Institute and Hospital. The study was approved by the Ethical Committee of Tianjin Medical University Cancer Institute and Hospital (Trial No. 11, 2020). All patients participating in this study provided signed informed consent. The colorectal tumor organoids culture was constructed according to a previous report.^[^ ^36^ ^]^

Tissue blocks were placed into a 60 mm dish with sterile forceps, cut into small pieces with surgical scissors and washed with ice‐cold PBS 3 times. Tissues were further washed with 10 mL Advanced DMEM/F12 and digested with 2 mg mL^−1^ collagenase (Sigma, C9407) at 37 °C for 12 h. After digestion, they were filtered through a 70 µm filter, resuspended twice with fresh medium containing 2% FCS and centrifuged at 400 rcf for 4 min. Single‐cell suspensions were collected and pelleted by centrifugation at 300 × *g* at 4 °C for 3 min. Subsequently, 1.1 ml of complete colorectal organoid growth medium (Cat# K2O‐M‐CO) and 120 µL of prechilled Matrigel (Corning Inc., Corning, NY, USA) were added. After thorough mixing, 0.3 mL of the mixture was plated in an ultralow adhesion culture plate (Costar) and incubated at 37 °C for 30 min to promote Matrigel solidification. Then, 300 µL of complete colorectal organoid growth medium was added to the well and kept at 37  °C in a 5% CO2 incubator. Dissociated cells were suspended in growth factor‐reduced Matrigel (Corning, Cat # 356 231) at 37 °C for 30 min. Next, the Matrigel was overlaid with 500 µL of complete human organoid medium (HOM), which was subsequently refreshed every other day. Culture media (300 µL) was added or changed every 3 days during the cultivation process. When at least 50% of organoids had reached a diameter of 200–500 µm, the organoids were passaged using TrypLE (Gibco, Grand Island, NY, United States). The PDOs were frozen using Recovery Cell Culture Freezing Medium (Gibco) and stored at −80 °C.

### Multiplex Fluorescent Immunohistochemistry Staining

Formalin‐fixed paraffin‐embedded CRC organoids were cut at a thickness of 5 µm onto slides. The slides were then baked at 65 °C for 2 h and immersed in xylene for 15 min in duplicate. The slides were then immersed in 100%, 95%, and 75% ethanol followed by water for 3 min. Then, antigen retrieval was performed with sodium citrate (pH 6.0). After washing with water, the slides underwent multiple rounds of multiplex fluorescent immunohistochemistry with the Opal Multiplex Fluorescent Immunohistochemistry kit (Perkin Elmer). The following primary antibodies and subsequent Opal TSA combinations were used: anti‐CD44 (1:250, abcam, ab254530) and Opal 520, anti‐ki67 (1:250, abcam, ab92742) and Opal 570, anti‐CEA (1:400, bioss, bs‐0719R) and Opal 620, anti‐EP‐CAM (1:250, Santa Cruz, sc‐53532) and Opal 690, and DAPI. The slides were mounted on coverslips with Prolong Diamond reagent (ThermoFisher).

### Establishment of Tumors in Mice

Male ob/ob mice (4 weeks old) were divided randomly into groups as described in Figure 9a. The prepared MC38 cells were suspended in 100 µL of serum‐free DMEM and injected subcutaneously into each ob/ob mouse (1 × 10^7^ cells per mouse). All mice were housed in pathogen‐free cages. All experimental procedures were approved by the Institutional Animal Care and Research Advisory Committee of Tianjin Medical University Cancer Institute and Hospital.

### Lentivirus Injection

For lentivirus injection, 5‐week‐old mice were anesthetized via intraperitoneal injections of avertin (1.2% 2,2,2‐ tribromoethanol, 25 µL g^−1^ body weight; Sigma‐Aldrich, USA). Mice were placed face up in the surgical field, and the area above the abdominal adipose tissue was sterilized with povidone iodine. The skin and peritoneum were incised 5–10 mm to expose the fat pad. The fat pad was shifted through the incision. The skin flap was opened with moist cotton swabs, and the abdominal fat pads were exposed. Then, the KD‐MTTP lentivirus (pHBLV‐sh‐Mttp‐Luc‐Puro) or control lentivirus was injected orthotopically into multiple distinct spots of the fat pads with an insulin syringe. The tissue was lifted during the injection to ensure that no virus was released. Then, the incisions were manually sutured. The procedure was repeated on the contralateral fat pad to complete the bilateral injection.

### Chemicals

Erastin (S7242), liproxstatin‐1 (S7699), Z‐VAD‐FMK (S7023), CQ (S6999) and necrostatin‐1 (S8251) were purchased from Selleck Chemicals. Oxaliplatin (O8390) and puromycin (P8230) were purchased from Solarbio. AA (90 010) was purchased from Cayman Chemical. All chemicals were stored at −20 °C.

### Statistical Analysis

All data were obtained from at least three independent experiments and were expressed as mean ± SD. No data were excluded in the experimental sections, and the exclusion criteria for the bioinformatics analysis are shown in the corresponding sections. Data from different treatment groups showed good homogeneity of variances. The exact sample size for each experimental group is shown in every Figure as the number of dots. Two‐tailed unpaired Student's *t* test was used to compare the differences between two different treatment groups, and one‐way ANOVA with post hoc tests were used to compare the differences among the three or more different treatment groups. All statistical analyses were accomplished using SPSS version 24.0 software (IBM) and GraphPad Prism software (v 7.0.0, GraphPad Inc., CA). *P* < 0.05 was considered statistically significant: *P < 0.05, **P < 0.01, and ***P < 0.001 and n.s. indicates no significant difference.

## Conflict of Interest

The authors declare no conflict of interest.

## Authors Contribution

Q.Z. performed most of the experiments, analyzed data and wrote the manuscript. T.D., H.Z., D.Z. and Q.Z. performed some of the experiments. M.B., R.L., T.N., and L.Z. reviewed and edited the manuscript. H.Z. and Z.Y. designed the experiments and edited the manuscript. Y.B. is the guarantor of this work and takes responsibility for the integrity of the data and the accuracy of the data analysis. All authors approved the final manuscript.

## Ethics Approval and Consent to Participate

Informed consent was provided by all patients, all aspects of this study were approved by the Ethics Committee of Tianjin Medical University Cancer Institute and Hospital. All animal procedures were performed in accordance with protocols approved by the Institutional Animal Care and Research Advisory Committee of Tianjin Medical University Cancer Institute and Hospital.

## Supporting information

Supporting Information

## Data Availability

Research data are not shared.
