# Supplementary material for: Adipocyte‐Derived Exosomal MTTP Suppresses Ferroptosis and Promotes Chemoresistance in Colorectal Cancer
Source: Adv Sci (Weinh). 2022 Aug 17;9(28):2203357. doi: 10.1002/advs.202203357 (PMC9534973; doi:10.1002/advs.202203357)
Supplement: Supplementary file 1 — Supporting Information [file ADVS-9-2203357-s001.pdf]

Figure S1, Supporting Information

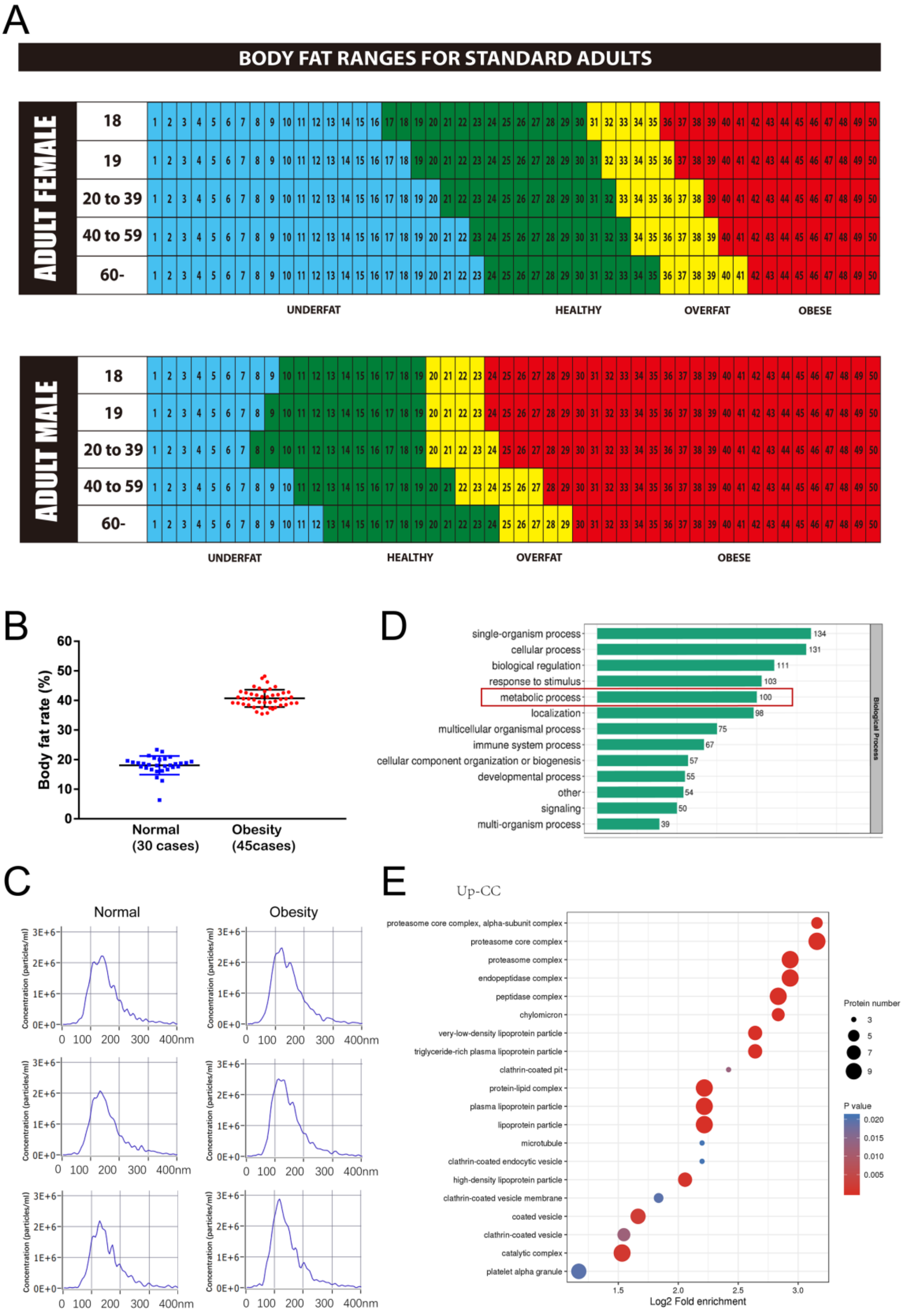

Figure legends

LC-MS/MS Analysis of plasma exosomes from normal weight and obese patients with CRC

(A) Body fat ratio ranges of adult female and adult male.

(B) Patients with CRC were divided into the normal weight group (n = 30) and obese group (n = 45) according to the body fat ratio.
(C) Size range of the plasma exosomes obtained from normal weight and obese patients with CRC detected using NTA.
(D) GO enrichment analysis of the main biological processes.
(E) KEGG pathway enrichment analysis of plasma exosomes from obese patients compared with control normal weight patients

Figure S2, Supporting Information

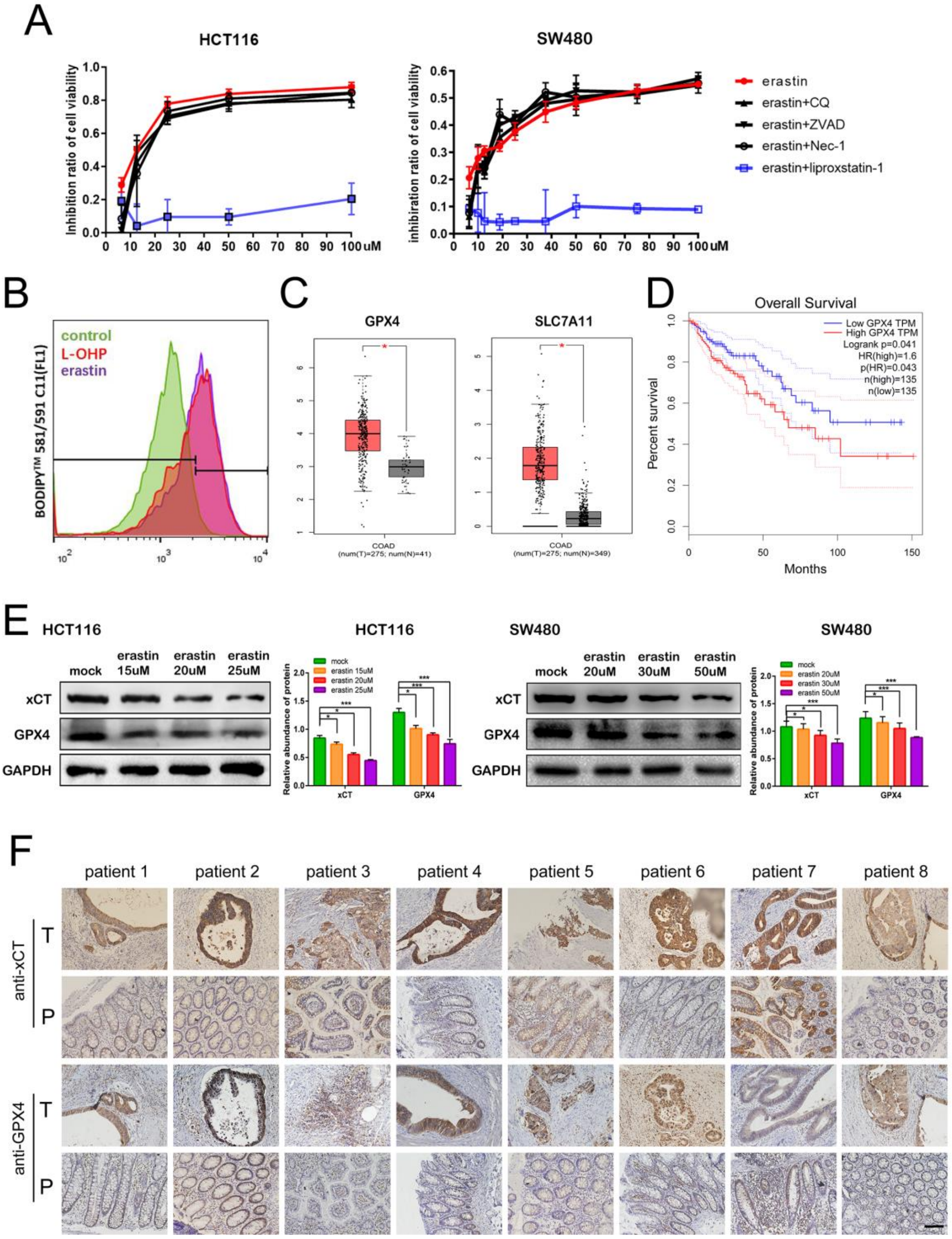

Figure legends

GPX4 and xCT are expressed at high levels in CRC and associated with a poor prognosis

(A) CRC cells were treated with erastin in the absence or presence of liproxstatin-1 (1 μM), Z-VAD-FMK (10 μM), CQ (1 μM) or necrosulfonamide (1 μM) for 24 h, and then cell viability was measured (n=3).

(B) Relative levels of C11-BODIPY in SW480 cells treated with erastin or L-OHP for 24 h (n = 3).

(C) Significantly higher levels of the GPX4 and xCT transcripts were detected in colorectal cancer tissues than in normal tissues in the GEPIA dataset. Means and SD are shown (*$P < 0.05$ as determined using *t* tests).
(D) The overall survival curve of patients with CRC stratified by GPX4 expression in the GEPIA dataset. Means and SD are shown (num (high) = 135, num (low) = 135, *$P < 0.05$ as determined using *t* tests).
(E) WB revealed the expression levels of xCT and GPX4 in CRC cells treated with gradient concentrations of erastin (n = 3).
(F) IHC analysis of GPX4 and xCT levels in paired paracarcinoma tissues and tumor tissues from patients with CRC (n=20). Scale bar = 200 μm.

Figure S3, Supporting Information

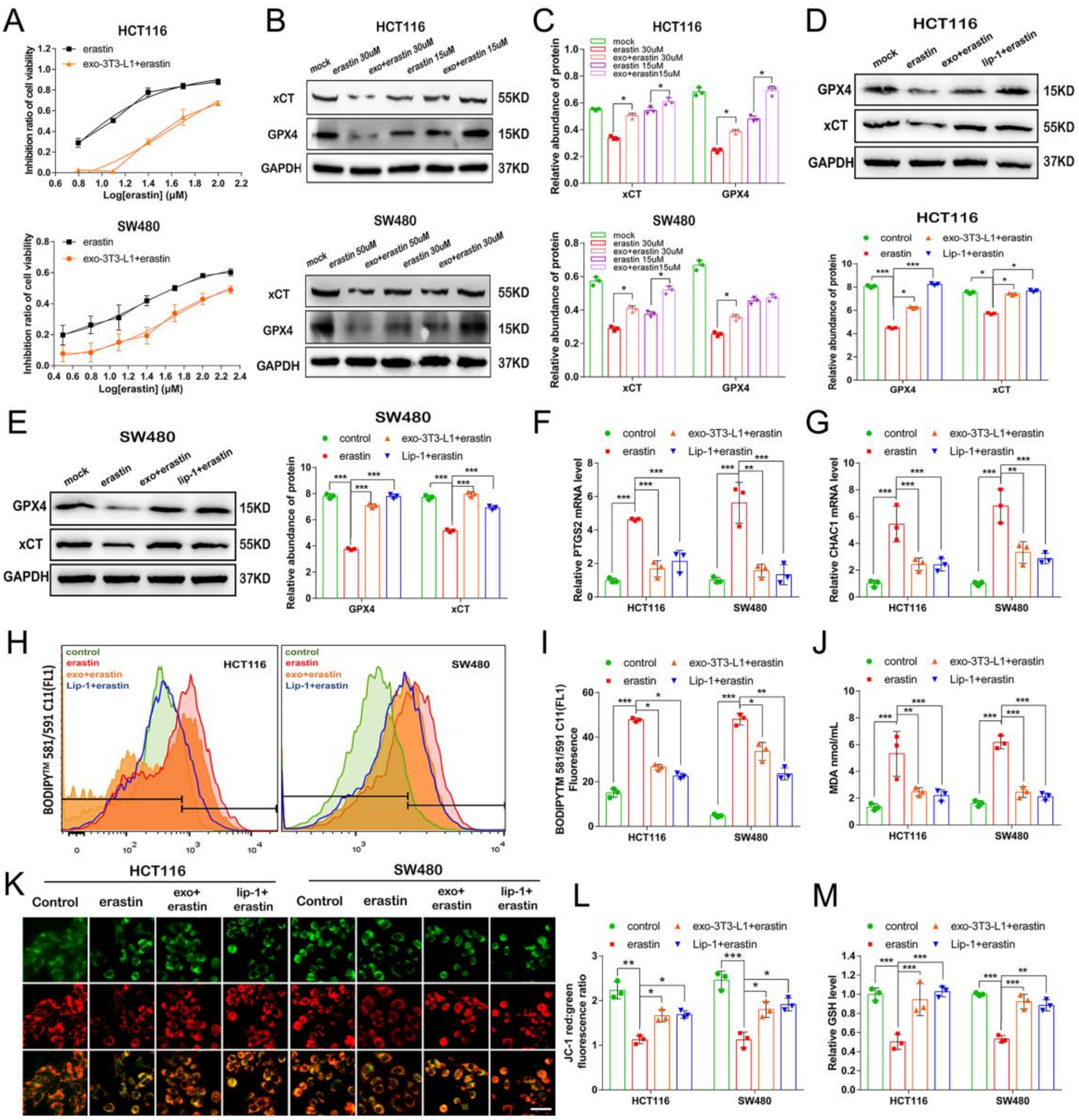

Figure legends

Adipocyte-secreted exosomes differentiation from 3T3-L1 preadipocytes reverse erastin-induced ferroptosis in CRC cells

(A) CCK-8 detection of the inhibition ratio of erastin-exposed CRC cells treated with or without mature adipose exosomes (n = 3).

(B) WB detection of xCT and GPX4 levels in HCT116 and sw480 cells treated with erastin or mature adipose exosomes following treatment with erastin (n = 3).

(C) Quantified analysis of (B) by gray scales. Error bars represent the standard deviation (SD) of the mean (n = 3), **P* < 0.05, as determined by the unpaired two-tailed Student's *t*-test.

(D, E) Levels of GPX4 and xCT protein levels in HCT116 (D) and SW480 cells (E) treated with erastin, pretreated with adipose exosomes or pretreated with lip-1 were determined using WB assay and quantified using gray scale analysis. Error bars represent the standard deviation (SD) of

the mean (n = 3), **P* < 0.05, ****P* < 0.001, as determined by the unpaired two-tailed Student's *t*-test.

(F, G) Relative levels of PTGS2 (F) and CHAC1 (G) mRNA expression in CRC cells treated with mature adipose exosomes or lip-1 following treatment with erastin for 24 h. Error bars represent the standard deviation (SD) of the mean (n = 3), *$P < 0.05$, **$P < 0.01$, ***$P < 0.001$, as determined by the unpaired two-tailed Student's *t*-test.

(H-M) Relative levels of C11-BODIPY (H, I), MDA (J), GSH (M) and JC-1 red: green fluorescence ratio (K, L) in control, lip-1 or mature adipocyte exosomes following treatment with erastin for 24 h (n = 3). Scale bars in (K)= 20 μm. Error bars represent the standard deviation (SD) of the mean (n = 3), *$P < 0.05$, **$P < 0.01$, ***$P < 0.001$, as determined by the unpaired two-tailed Student's *t*-test.

Figure S4, Supporting Information

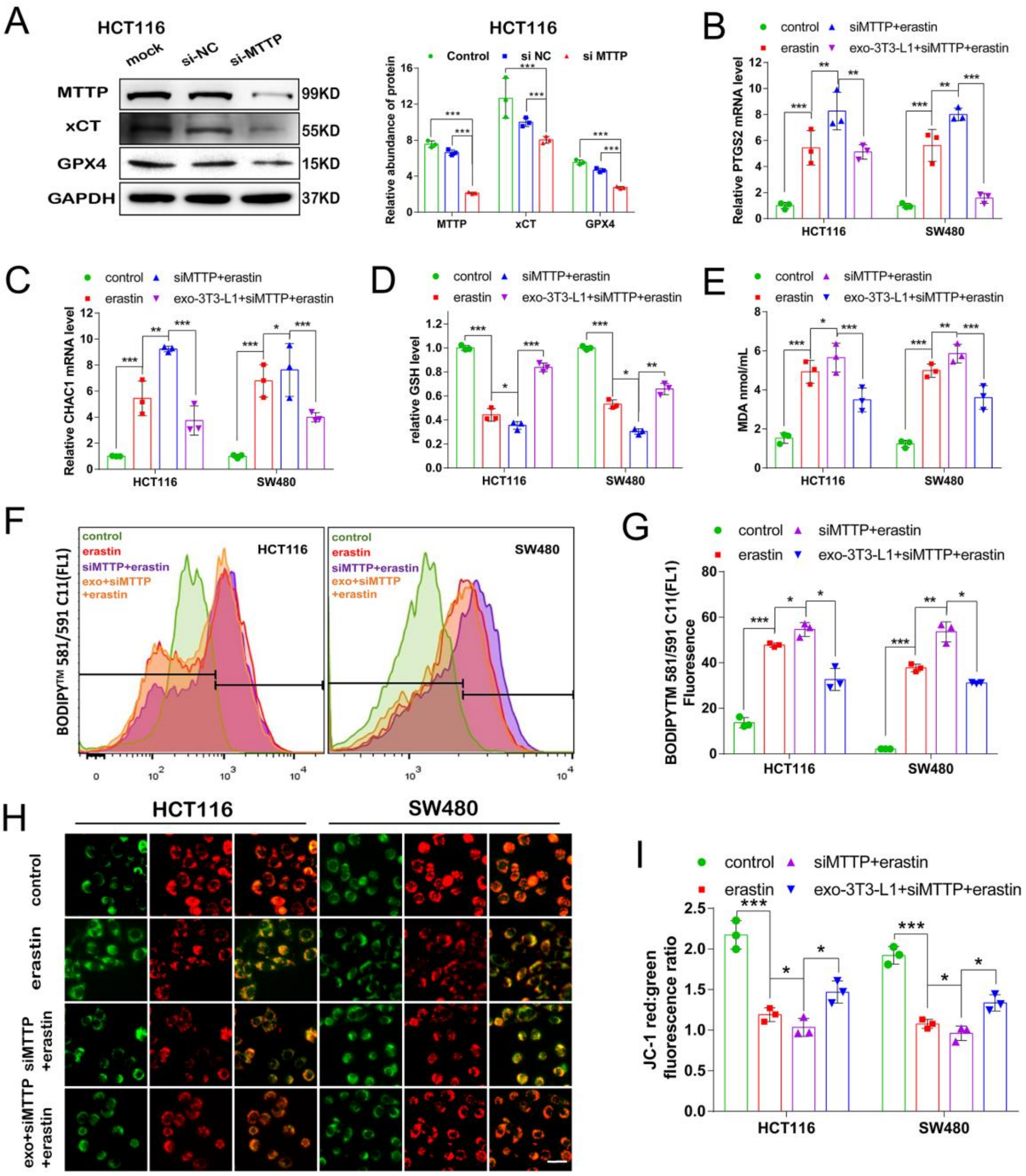

Figure legends

Inhibition of MTTP promotes ferroptosis in colorectal cancer cells.

(A) WB revealed the expression levels of MTTP, xCT and GPX4 in HCT116 cells with MTTP knockdown and quantified using a gray scale analysis. Error bars represent the standard deviation (SD) of the mean (n = 3), ***$P < 0.001$, as determined by the unpaired two-tailed Student's *t*-test.

(B, C) Relative mRNA expression levels of PTGS2 (B) and CHAC1 (C) in CRC cells with MTTP knockdown or treated with mature adipose exosomes following treatment with erastin for 24 h. Error bars represent the standard deviation (SD) of the mean (n = 3), *$P < 0.05$, **$P < 0.01$, ***$P < 0.001$, as determined by the unpaired two-tailed Student's *t*-test.

(D-I) Relative levels of GSH (D), MDA (E), C11-BODIPY (F, G) and JC-1 red: green fluorescence ratio (H, I) in control, MTTP knockdown pretreated or adipose exosome pretreated cells following treatment with erastin for 24 h. Scale bars in (M)= 20 μm. Error bars represent the standard deviation (SD) of the mean (n = 3), *$P < 0.05$, **$P < 0.01$, ***$P < 0.001$, as determined by the unpaired two-tailed Student's *t*-test.

Figure S5, Supporting Information

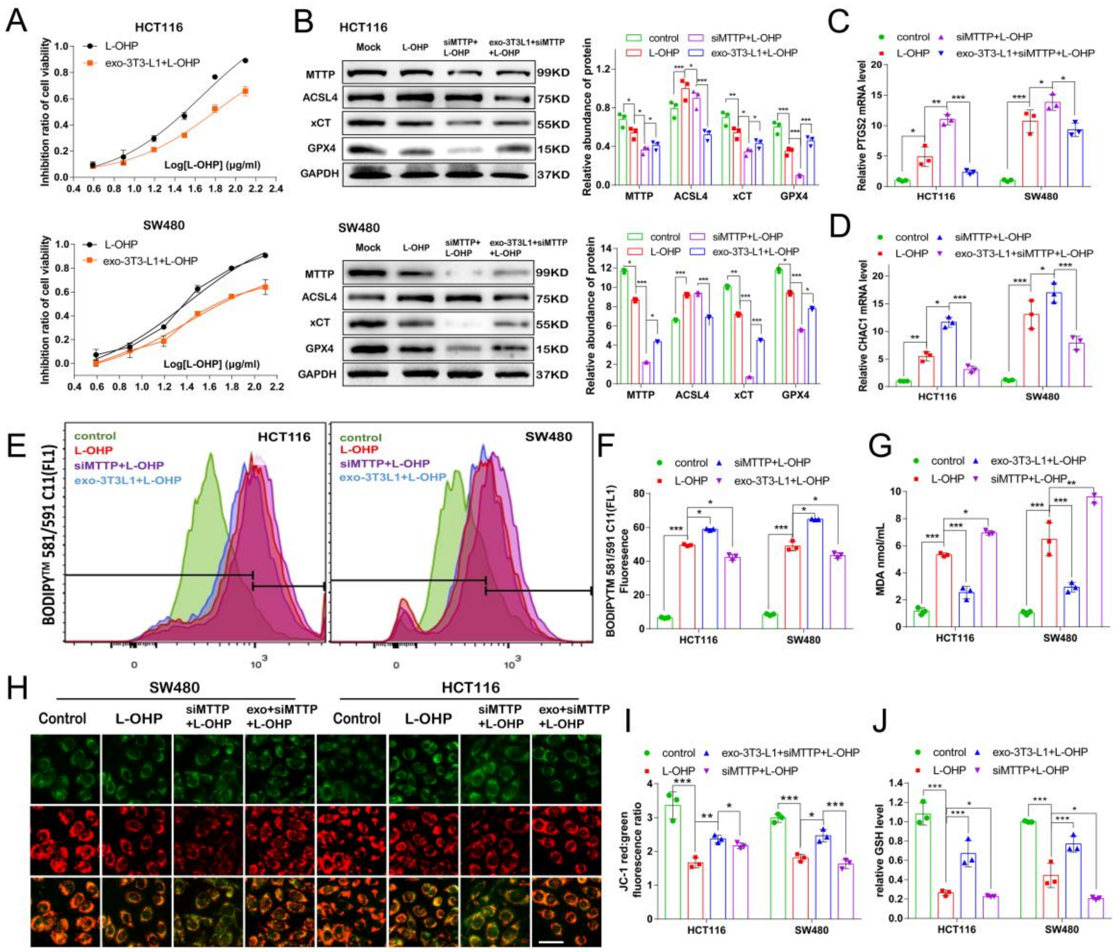

Figure legends

Adipose exosomes differentiation from 3T3-L1 preadipocytes reduce the sensitivity of CRC cells to L-OHP

(A) CCK-8 detection of the inhibition ratio of L-OHP in CRC cells treated with or without mature adipose exosomes (n = 3).

(B) Effects of the MTTP siRNA and mature adipocyte exosomes on MTTP, ACSL4, xCT and GPX4 protein levels in CRC cells treated with L-OHP and quantified by gray scale analysis. Error bars represent the standard deviation (SD) of the mean (n = 3), **P* < 0.05, ***P* < 0.01, ****P* < 0.001, as determined by the unpaired two-tailed Student's *t*-test.

(C, D) Relative levels of PTGS2 (C) and CHAC1 (D) mRNA expression in CRC cells treated with mature adipocyte exosomes or the MTTP siRNA following treatment with L-OHP were examined. Error bars represent the standard deviation (SD) of the mean (n = 3), **P* < 0.05, ***P* < 0.01, ****P* < 0.001, as determined by the unpaired two-tailed Student's *t*-test.

(E-J) Relative levels of C11-BODIPY (E, F), MDA (G), GSH (J) and JC-1 red: green fluorescence ratio (H, I) in control, MTTP knockdown pretreated or adipose exosome pretreated cells following treatment with L-OHP. Scale bar in (J)= 20 μm. Error bars represent the standard deviation (SD) of the mean (n = 3), **P* < 0.05, ***P* < 0.01, ****P* < 0.001, as determined by the unpaired two-tailed Student's *t*-test.
